# The Role of Extracellular Vesicles in the Hallmarks of Cancer and Drug Resistance

**DOI:** 10.3390/cells9051141

**Published:** 2020-05-06

**Authors:** Cristina P. R. Xavier, Hugo R. Caires, Mélanie A. G. Barbosa, Rui Bergantim, José E. Guimarães, M. Helena Vasconcelos

**Affiliations:** 1 i3S—Instituto de Investigação e Inovação em Saúde, Universidade do Porto, 4200-135 Porto, Portugal; cristinax@ipatimup.pt (C.P.R.X.); hcaires@ipatimup.pt (H.R.C.); melanieb@ipatimup.pt (M.A.G.B.); rbergantim@ipatimup.pt (R.B.); jeteguimaraes@gmail.com (J.E.G.); 2 Cancer Drug Resistance Group, IPATIMUP—Institute of Molecular Pathology and Immunology, University of Porto, 4200-135 Porto, Portugal; 3 Clinical Hematology, Hospital São João, 4200-319 Porto, Portugal; 4 Clinical Hematology, FMUP-Faculty of Medicine, University of Porto, 4200-319 Porto, Portugal; 5 Cooperativa de Ensino Superior Politécnico e Universitário, CESPU, 4585-116 Gandra, Paredes, Portugal; 6 Department of Biological Sciences, FFUP-Faculty of Pharmacy, University of Porto, 4050-313 Porto, Portugal

**Keywords:** extracellular vesicles, hallmarks of cancer, tumor microenvironment, cancer drug resistance

## Abstract

Extracellular vesicles (EVs) mediate intercellular signaling and communication, allowing the intercellular exchange of proteins, lipids, and genetic material. Their recognized role in the maintenance of the physiological balance and homeostasis seems to be severely disturbed throughout the carcinogenesis process. Indeed, the modus operandi of cancer implies the highjack of the EV signaling network to support tumor progression in many (if not all) human tumor malignancies. We have reviewed the current evidence for the role of EVs in affecting cancer hallmark traits by: (i) promoting cell proliferation and escape from apoptosis, (ii) sustaining angiogenesis, (iii) contributing to cancer cell invasion and metastasis, (iv) reprogramming energy metabolism, (v) transferring mutations, and (vi) modulating the tumor microenvironment (TME) by evading immune response and promoting inflammation. Special emphasis was given to the role of EVs in the transfer of drug resistant traits and to the EV cargo responsible for this transfer, both between cancer cells or between the microenvironment and tumor cells. Finally, we reviewed evidence for the increased release of EVs by drug resistant cells. A timely and comprehensive understanding of how tumor EVs facilitate tumor initiation, progression, metastasis and drug resistance is instrumental for the development of innovative EV-based therapeutic approaches for cancer.

## 1. Introduction

**Figure 1:**
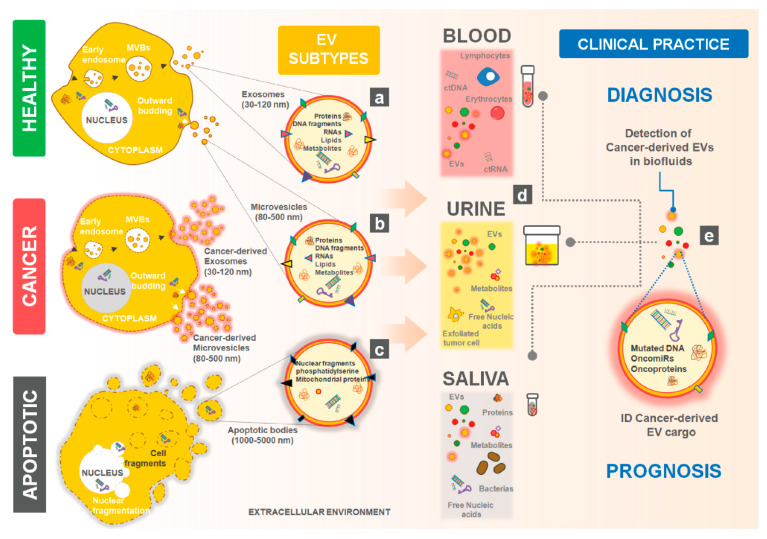
Features of Extracellular Vesicle (EV) sub-populations. EVs are comprised of a heterogeneous group of lipid membrane enclosed vesicles produced by virtually all cells of the organism. EVs play a key role in intercellular communication to support homeostasis or cancer progression. Importantly, this heterogeneous group of EVs may include (**a**) exosomes with a size range between 30–120 nm that originate via the endosomal system, (**b**) microvesicles with a size range between 80–500 nm that derive from the outward budding of the cells’ plasma membrane and even (**c**) apoptotic bodies that are secreted during the fragmentation of apoptotic cells. Upon secretion to the extracellular environment, exosomes and microvesicles have overlapping size range and share many markers (e.g., CD63, HSP70, CD9, CD81) while apoptotic bodies are characterized by an enrichment of phosphatidylserine on their surface. The similar features between exosomes and microvesicles make an accurate discrimination of EV origin very difficult when these subpopulations are mixed in complex biofluids. (**d**) The cancer-derived EVs are highly abundant in biological fluids such as blood, urine and saliva, and their surface immunophenotypic protein markers reflect the cell of origin. In clinical practice, (**e**) the detection of cancer-derived EVs in the biological fluids of patients can be explored for the disease diagnosis, while the EV cargo characterization also provides important clues on the disease prognosis.

Extracellular vesicles (EVs) are cell-released particles ranging in size from 30 to 1000 nm, enclosed within a phospholipid bilayer and which do not replicate. In addition to managing cellular waste, they play a pivotal role in mediating intercellular communication under both physiological and pathological conditions [1,2,3,4]. EVs can vary in size, properties, and biological functions and are mostly classified as exosomes (30–120 nm in diameter), microvesicles (also known as MVs, ectosomes or microparticles, 100–1000 nm), or apoptotic bodies (ranging from 800 to 5000 nm) (Figure 1a–c). Their classification depends on their biogenesis pathway. Exosomes are derived from the endolysosomal pathways, which involves inward budding in early endosomes, forming multivesicular bodies. Compared to other classes, exosomes seem to represent a more heterogeneous population of EVs. On the other hand, microvesicles are released via direct shedding from the plasma membrane. Finally, apoptotic bodies are formed during cytoskeletal rearrangement, being released by outward blebbing and decomposition of the cell membrane of apoptotic cells. Among them, apoptotic bodies are less frequently involved in intercellular communications [2,5,6].

EVs were originally reported in 1946 by Chargaff and West as procoagulant platelet-derived particles in normal plasma [7]. Later this observation was termed as ‘‘platelet dust’’ by Wolf in 1967 [8]. The confusion on the use of the right nomenclature arose from the lack of standardization of both isolation procedures and methods for the proper characterization of EV subgroups. Thus, it was only in 2011 that an important step was taken to unify the nomenclature and the methodologies for isolating EVs.

Indeed, given the difficulty in experimentally supporting the attribution of certain activities to EV subtypes, the absence of consensus in the nomenclature and the limited knowledge on their molecular mechanisms of biogenesis and release, recent guidelines from the International Society for Extracellular Vesicles (ISEV: www.isev.org/) were published. These guidelines advise researchers to refer to EVs by their physical characteristics (size and/or density), biochemical composition or descriptions of the cell of origin, unless their biogenesis pathway is confirmed, for example through live imaging techniques [1]. Therefore, in this review, we will use the widely adopted generic term “extracellular vesicles” (EVs) to refer to exosomes and/or microvesicles. 

EVs are produced by almost all organisms and cell types and allow the intercellular transfer of molecules such as mRNAs, lipids, metabolites, proteins and non-coding RNAs (such as microRNAs and long non-coding RNAs), and even DNA fragments, which can induce phenotypic reprogramming of recipient cells [1,2,3,4,9,10,11,12,13,14,15] (Figure 1d). Tumor-derived EVs have been shown to educate recipient cells towards a tumor-promoting phenotype, being involved in multiple steps of cancer development with roles in all cancer hallmarks. Tumor microenvironment (TME) may be altered by EVs, to support tumor growth and survival, induce angiogenesis, tumor cell invasion and metastasis, initiate resistance to cell death, evade immune response, reprogram cellular energy metabolism and drug resistance [3,16,17,18,19,20].

EVs can be recovered from common body fluids, such as blood and plasma [21,22], semen [23], urine [24], breast milk [25], nasal secretions [26], saliva [27], and even fecal matter [28] (Figure 1e). Since their content reflects their cell of origin, EVs are attractive as potential biomarkers (or source of biomarkers) of certain malignancies, including cancer [2,29,30]. However, the heterogeneity of the EV populations within a biological sample might complicate such biomarker analysis, particularly since a single cell is capable of producing different subtypes of these vesicles [2,31]. Furthermore, there is a lack of protocols to permit single EV analysis with a high degree of purity and specificity [1,2].

EVs encapsulation of their cargo into a phospholipid bilayer provides this cargo with greater stability, a longer half-life, higher resistance to degradation, and a greater ability to travel long distances when compared to free proteins, lipids and nucleic acids in the cytoplasm. Moreover, EVs have the ability to transfer their cargo to recipient cells and organs that are protected by physiological barriers, such as the blood-brain barrier [32,33,34]. Interestingly, although there is no consensus, some studies show that cancer cells produce more EVs than normal cells [13,35]. However, others report the lack of statistical difference in the levels of plasma EVs between cancer patients and healthy people [19,36]. This conflicting evidence might be attributed to a low inter-laboratory reproducibility and to non-standardization of isolation and purification protocols for EVs [13].

Nevertheless, accumulating evidence suggests that tumor-derived EVs pay an important role in all steps of cancer progression, which makes them attractive for developing EV-based liquid biopsies for cancer patients (Figure 1f). Compared to conventional biopsies they: (1) are a simpler and less invasive method for early diagnosis, (2) facilitate surveillance of the patient’s cancer stage and treatment efficacy, and (3) offer surveillance of patient relapse and prognosis [29,37,38,39]. However, some problems may have to be addressed when considering implementing EV-based liquid biopsies in the clinic, such as the time required for analysis, high costs involved, or need to find a compromise between high specificity and high sensitivity [39]. Additionally, the EVs retrieved from liquid biopsies are not necessarily representative of the most abundant cell type in a certain tissue [40]. Additionally, the EVs’ populations, cargo levels, and selective packaging are highly heterogeneous and changeable under different physiological conditions, internal cellular processes or external stimuli (such as temperature and stress), thus offering a mere snapshot of the molecular circumstances in their cell of origin at their point of release from cells [2,41,42]. In addition, the isolation, purification, and characterization of EVs as well as the separation of EV subpopulations, still requires standardization. To this end, many novel methods are under development varying in cost, sensitivity, purity and time required [1,2,33,43]. Selection of the purification method to be used should be based on the sample type and amount as well as the downstream analysis to be performed [1,33]. Additionally, specific methods might bias the retrieval of certain subpopulations of EVs [43].

EVs are also being considered for cancer therapy, either as cancer vaccines or as drug delivery systems to accumulate certain drugs or siRNAs at specific tumor sites, while reducing toxicity-related adverse effects [44,45,46,47]. 

The following section reviews recent findings on the association of cancer-derived EVs with the well-known cancer hallmarks and with cancer drug resistance.

## 2. Tumor-Derived EVs affecting Cancer Hallmarks

**Figure 2:**
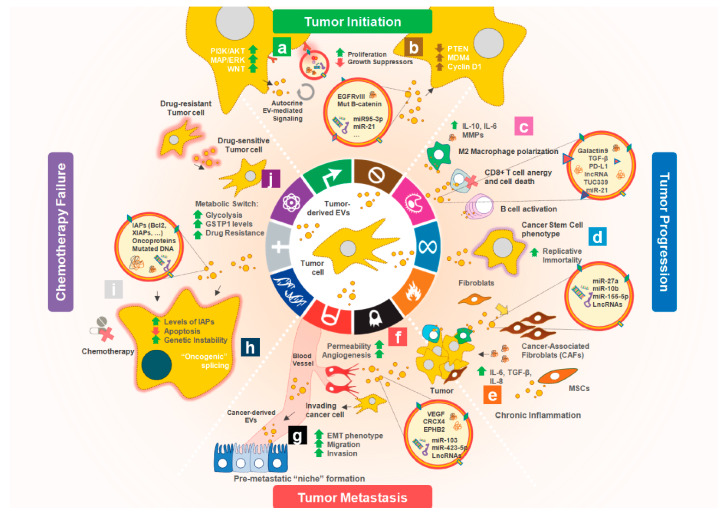
Impact of tumor-derived EVs on the acquisition and maintenance of cancer hallmarks traits. From the onset of tumor initiation, cancer-derived EVs modulate the phenotype of multiple recipient cell types to support tumor progression, metastasis and resistance to therapy. Indeed, many of the cancer cell clones may rely on the shedding of cancer-derived EVs to enable the (**a**) activation of several tyrosine kinase receptors and their downstream signalling pathways (e.g., MAP/ERK, PI3K/AKT and/or WNT). This cancer EV-mediated sustained proliferative signalling can be either autocrine or paracrine and confers tumor cells a key proliferative advantage. Moreover, in a synergistic event, (**b**) cancer-derived EVs also carry many oncoproteins and oncomiRs that when internalized by target recipient cancer cells enable them to override the growth suppressor signalling (e.g., through the reduction of PTEN and exacerbated expression ofMDM4 and/or cyclin D1 “oncogenic” splicing variants). Importantly, for successful tumor progression, tumor cells must acquire the ability to (**c**) evade immune destruction. Cancer-derived EVs serve this purpose as vectors for many immunosuppressive molecules, including galactin9 which binds TIM-3 on T cells inducing their death. Additionally, EV-associated TGF-β, PD-L1 and several miRs induce an immunosuppressive phenotype when internalized by immune cells. This includes the induction of a M2-like secretion profile on macrophages, CD8+ T cells anergy or stimulation of B and T cells to secrete a wide array of tumor supporting cytokines. (**d**) Cancer-derived EVs cargo may include TERT mRNA and/or other non-coding RNAs that induce the expression of telomerase in recipient fibroblasts and in other mutated cell clones, enabling a cancer stem cell phenotype with the acquisition of replicative immortality. (**e**) Cancer-derived EVs are also an important player for the perpetuation of chronic inflammation within the tumor microenvironment, which fosters multiple hallmark functions. Cancer-derived EVs carry several miRs including miR-27, -10b, -155-5p and other LncRNAs that target nearby fibroblasts, transforming them into cancer-associated fibroblasts (CAFs). In turn, CAFs secrete high amounts of IL-6 and TGF-β to the tumor microenvironment. Many of these cancer-derived EVs can also “educate” nearby Mesenchymal Stromal Cells (MSCs) to secrete large amounts of IL-8 and other immunosuppressive cytokines. Interestingly, this inflammatory microenvironment is prone to promote the formation of new blood vessels towards the tumor. (**f**) In fact, cancer-derived EVs cargo may also include VEGF, CRCX4 and EPHB2 and other epigenetic modulators, such as miR-103 as well as other lncRNAs, that increase the permeability of nearby blood vessels recruiting endothelial tip cells to promote angiogenesis. (**g**) Many of these EVs carry Matrix Metalloproteases (MMPs) and upon internalization by nearby cancer cells activate an Epithelial to Mesenchymal Transition (EMT) phenotype, inducing tumor cell invasion and metastasis to distant organs. Simultaneously, cancer-derived EVs will act on distant tissues to increase the expression of specific integrins and establish the pre-metastatic “niche”. Noteworthy, the specific cancer-derived EVs tropism seems to be heavily reliant on the origin of the primary tumor. Moreover, the cancer-derived EVs cargo may in some cases include fragments of mutated DNA and other oncoproteins (**h**) that when transferred to other cancer cells will increase their genome instability and in turn generate high genetic diversity. Interestingly, (**i**) EVs may allow the horizontal transfer of drug resistance phenotype from drug resistant cancer cell clones to sensitive ones, mediated by cargo such as proteins (e.g., antiapoptotic proteins or drug efflux pumps), miRNAs, mRNAs, lncRNAs or lipids. (**j**) The same cancer drug-resistant derived EVs will induce a metabolic switch in recipient drug-sensitive cancer cells, reprogramming the energy metabolism towards glycolysis and increasing their levels of detoxifying enzymes such as Glutathione S-transferase P (GSTP1) granting a multidrug resistant phenotype in these cells.

As mentioned above, EVs released by some cells (the donor cells) can be taken up by other cells (the recipient cells) where they release their cargo, thus being responsible for intercellular communication [3]. Several studies have revealed the remarkable impact of tumor-derived EVs on the well-known cancer hallmarks, ultimately interfering with cancer progression and drug resistance. Indeed, the effect of tumor-derived EVs on sustained cellular proliferation and resistance to cell death, induction of angiogenesis, promotion of invasion and metastasis, reprogramming of cellular energy metabolism, evasion from immune response, transfer of mutations, and modulation of the TME underpins their relevance as central mediators in key cancer processes (Figure 2).This will be reviewed in the following sections, together with the EVs cargo derived from different types of cancers and identified to have a biological relevance (Table 1).

### 2.1. Promoting Cell Proliferation and Escape from Apoptosis

EVs released by cancer cells can support the enrolment of normal cells into the tumorigenic process, facilitating their phenotypical transformation and thus contributing to tumor growth by promoting proliferative signaling. Additionally, EVs can support cancer cells escape from apoptosis. For example, a study [48] showed that the splicing factor RBM11 present in EVs released by glioblastoma cell lines was transferred to recipient tumor cells and induced the splicing of MDM4 and Cyclin D1 into a more oncogenic isoform, contributing to an increase in survival and impairing apoptosis. In addition, in vivo work in mice injected with EVs from glioblastoma cells confirmed the potential of those EVs in promoting malignancy in recipient tumor cells [48]. Another study demonstrated that EVs released by glioblastoma cells (either cell lines or patient-derived cancer stem cells) mediated the intercellular transfer of the protein chloride intracellular channel-1 (CLIC1), supporting the growth of other (recipient) glioblastoma cells. In the same study, the injection of glioblastoma cells with EVs containing CLIC1 into nude mice enhanced tumor growth when compared with EVs not containing CLIC1 [49]. In melanoma, the transfer of PDGFR-β mediated by EVs released by melanoma (donor) cells caused an activation of the PI3K/Akt pathway and escape from the MAPK pathway on BRAF mutated (recipient) cells, contributing to cellular proliferation and inhibition of apoptosis [50]. In fact, the activation of the PI3K/AKT and MAP/ERK pathways mediated by EVs was also verified in other cancer cell types. For instance, EVs released by bladder and gastric cancer cells promoted cell proliferation and inhibited apoptosis of recipient cancer cells through the activation of both pathways [116,117]. 

Other mechanisms contributing to the promotion of tumor cell proliferation mediated by EVs were also described such as the intercellular transfer between pancreatic cancer cells of the zinc transporter ZIP4, leading to enhancement of tumorigenesis. In vivo, EVs released by pancreatic cancer cells injected into a nude mouse model promoted a more rapid and larger tumor growth than EVs from those cells that had been knocked down for ZIP4 [51]. Moreover, colon cancer derived EVs containing high levels of N-terminal truncated isoforms of p73 (DNp73) mRNA stimulated the growth of recipient cells. This result was subsequently confirmed in vivo using mice injected with EVs from DNp73 overexpressing cells, giving rise to greater tumor size when compared to mice injected with EVs from control cells [56]. 

The relevance of microRNAs (miRNAs) present in the cargo of EVs on cancer cell proliferation and apoptosis has also been verified in several studies. For instance, EVs shed by esophageal cancer cells transfer miR-93-5p to recipient neighbour cancer cells, affecting PTEN expression and its downstream proteins, p21 and cyclin D1, thus increasing cell proliferation of recipient cells [52]. Other examples are those of miR-1246 (which suppresses clyclin-G2 (CCNG2) levels) on EVs shed by breast cancer cells [54] and miR-205 on EVs shed by cholangiocarcinoma cells [55], which impacted cancer cell proliferation. Moreover, EVs shed by colon cancer cells containing high levels of miR-193a and mir-200b were responsible for the promotion of colon cancer cellular proliferation. Further in vivo work proved the impact of miR-193a and miR200b on tumor progression, using tumor-bearing nude mice or tumor xenografts, respectively, injected with EVs containing the respective miRNAs [57,58]. EVs released by pancreatic cancer cells in vitro transferred miR-23b-3p [53] and miR-222 [118] to neighboring cancer cells in order to promote cell proliferation. Interestingly, the miR-23b-3p was isolated from the plasmatic EVs of pancreatic cancer patients [53]. Remarkably, miR-222 and miR-146b were found to be overexpressed in EVs released by papillary thyroid cancer cells, being responsible for cancer cell proliferation in vitro [60]. The involvement of miRNAs from EVs released by gastric cancer cells on cancer cell proliferation via CD97-associated pathways, such as the MAPK signaling pathway, was also observed [119]. EVs containing miR-21 from ovarian serous carcinoma cells also contributed to malignant transformation and progression, through post-transcriptional inhibition of the tumor suppressor programmed cell death 4 (PDCD4) [61]. In leukemia, EVs isolated from patient samples had a specific miRNA signature when compared to EVs from healthy donors. This suggests that miRNAs carried by EVs are likely to be involved in the tumorigenesis of this cancer type [62]. 

Long non-coding RNAs (lncRNA) present on EVs released by cancer cells also have a role in cancer progression and apoptosis. Indeed, various lncRNAs found in the cargo of EVs released by many tumor cell lines favoured cancer cell growth, including the lncRNA ZFAS1 in EVs released by gastric cancer cells [63], lncRNA PVT1 in EVs released by colon cancer cells [59], lncRNA ZEB1-AS1 in EVs shed by esophageal cancer cells [64] and lncRNA TUC339 in EVs released by hepatocellular carcinoma cells [65]. Remarkably, the clinical impact of the lncRNA ZEB1-AS1 was observed by its detection at higher levels in EVs isolated from esophageal cancer patients when compared to EVs from healthy individuals [64].

### 2.2. Sustaining Angiogenesis

The development of de novo vasculature is essential for tumor growth and metastasis and EVs are key players in this process. Indeed, the ability of tumor-derived EVs to sustain angiogenesis by promoting communication between cancer cells and endothelial cells has been described. Several studies report the effect of EVs released by glioma cells on angiogenesis. In these studies, the angiogenesis process was enhanced via upregulation of the vascular endothelial growth factor (VEGF) on recipient endothelial cells which was caused by the presence of promoting-angiogenic factors on the cargo of EVs, such as lncRNA CCAT2 [66], lncRNA POU3F3 [67], miR-21 [68] or CXCR4 receptor [69]. The VEGF has also been detected in the cargo of EVs and was found to contribute to angiogenesis stimulation [68,69]. Moreover, EVs from head and neck squamous cell carcinoma also exerted an effect, both in vitro and in vivo, in driving tumor angiogenesis through the ephrin type B receptor 2 (EPHB2) [70]. Another study showed that EVs released by hepatocellular carcinoma cells promoted endothelial cell migration in vitro, through vasorin transfer which plays a role in vasculogenesis [71]. EVs shed by multiple myeloma cells carry a piwi-interacting RNA (the piRNA-823, that belongs to a class of small non-coding RNAs), which is able to re-educate endothelial cells towards an environment amenable to the growth of multiple myeloma cells, by enhancing VEGF, interleukin 6 (IL-6) and ICAM-1 (intercellular adhesion molecule 1) expression. In addition, EVs carrying piRNA-823 promoted the growth of xenograft tumors in vivo [73]. Furthermore, miR-25-3p secreted via EVs shed by colon cancer cells was delivered to vascular endothelial cells, disrupting the integrity of endothelial barriers, and thus inducing vascular permeability and angiogenesis. An induction of the pre-metastatic niche formation was also observed when EVs derived from colon cancer cells containing miR-25-3p were injected into nude mice [74]. Similarly, hepatocellular carcinoma cells secreted EVs containing miR-103 which attenuated the endothelial junction integrity by directly inhibiting the expression of VE-Cadherin, p20-catenin, and zonula occludens 1, thus increasing vascular permeability [72]. Moreover, in lung cancer, the intercellular transfer mediated by EVs of miR-14-3p and miR-145-5p from cancer cells to endothelial cells, led to an increase of tube formation by endothelial cells and thus promoted angiogenesis [75]. Also, EVs from epithelial ovarian cancer cells containing miRNA-141-3p promoted endothelial cell angiogenesis through activation of the JAK/STAT3 and NF-κB signaling pathways [77]. Another group also demonstrated in vitro that ovarian cancer cell-derived EVs induced the expression of VEGF in endothelial cells, thus influencing their vascular behaviour [120]. Moreover, EVs shed by oral cancer cells contained miR-142-3p that could be taken up by recipient endothelial cells, promoting angiogenesis mediated by the protein TGFBR1. Increased vascular density by miR-142-3p was confirmed in vivo using miR-142-3p overexpression in the mouse xenograft model of oral cancer [78]. Curiously, EVs released by lung cancer cells during radiation therapy had increased levels of miR-23a which promoted endothelial cell angiogenesis, suggesting that this could be a resistance mechanism to this type of therapy [76]. 

Of note however, are studies reporting contradictory effects of EVs on angiogenesis. For example, in nasopharyngeal carcinoma, EVs containing miR-23a induced angiogenesis by directly targeting the testis-specific gene antigen (TSGA10) [79] while EVs containing miR-9 were anti-angiogenic by regulating the PDK/Akt pathway [121]. The levels of miR-23a on EVs isolated from the serum of metastatic nasopharyngeal carcinoma patients were higher than on EVs derived from healthy volunteers [79]. Likewise, EVs shed by oral squamous carcinoma cells also presented pro- or anti-angiogenic properties, depending on the invasive capacity of the cells [122].

### 2.3. Contributing to Cancer Cell Invasion and Metastasis

Several studies have demonstrated the involvement of EVs in tumor metastasis through the stimulation of cancer cell migration and invasion. For example, some studies underline the role of EVs shed by prostate carcinoma cells in enhancing cell motility and metastatic potential of cancer cells and in inducing malignant features in normal prostate cells. The mechanisms proposed include the induction of mesenchymal traits in non-tumor cells [123], modulation of the androgen receptor and TGF-β signaling [124], or the alteration in cellular levels of tetraspanins CD9 and CD151 [125] in the recipient cancer cells. 

The horizontal transfer mediated by EVs of molecules responsible for enhancing cellular migration and facilitating invasion of recipient cancer cells was also observed in other cancer cell types. These include in metastatic breast cancer cells through Caveolin-1 transfer [80], in colon cancer cells by Wnt5b protein transfer [81] and in hepatocarcinoma cell lines through the transfer of CXC chemokine receptor 4, CXCR4 [84] or SMAD3 [83,85]. In fact, the clinical value of SMAD3 was confirmed by its presence at high levels in EVs isolated from hepatocellular carcinoma patients when compared to EVs isolated from patients with benign hepatomas or from healthy donors [85]. In renal carcinoma, EVs shed by cancer cells inhibited hepaCAM (a cell adhesion molecule) in a p-Akt dependent manner, thus promoting malignancy by increasing cell migration [126]. Interestingly, the status of KRAS was found to be essential for the ability of colon cancer cells to invade. EVs derived from malignant donor cells with the KRAS mutant allele have high amounts of amphiregulin (AREG), which is a ligand for EGFR, increasing the potential invasiveness and metastasis in recipient cells [82]. Moreover, colon cancer cell EVs were also shown to induce hepatocellular cancer cells migration via activation of the MAPK/ERK pathway in recipient cells [127].

The role of miRNAs from the cargo of EVs on recipient cancer cells invasion and migration has also been described in vitro. One example includes the identification of miR-205-5p or miR-423-5p in EVs released by cholangiocarcinoma or by gastric cancer cells respectively [55,87]. In vivo studies also demonstrated that the injection of EVs enriched in miR-423-5p into xenografts increased the number of metastatic tumor nodes. At a clinical level, higher expression of miR-423-5p was detected in EVs isolated from the serum of gastric cancer patients than in EVs isolated from healthy volunteers [87]. Additionally, in EVs shed by hepatocellular carcinoma cancer cell lines, miR-93 [86] and miR-103 [72] were also found to contribute to cancer cell invasiveness. Furthermore, higher levels of miR-93 were observed in EVs isolated from hepatocellular carcinoma patients than from healthy controls, related with a large tumor size and later tumor stage [86]. Moreover, the presence of miR-103 in EVs derived from tumor-bearing mice was associated with increased vascular permeability and tumor metastasis [72]. In another study, EVs shed by prostate carcinoma cell lines contained miR-1246 that inhibits N-cadherin and vimentin activities, which then inhibited epithelial-mesenchymal transition (EMT). The miR-1246 was detected in EVs isolated from the serum of xenograft mouse models and from the serum of aggressive prostate cancer patients [88]. Other work reported that EVs isolated from the serum of glioblastoma patients encapsulated increased levels of miR-148a. This targeted the cell adhesion molecule 1 (CADM1) when compared to EVs isolated from healthy volunteers, suggesting that miR-148a might be involved in cancer invasion [89]. The presence of miR-99a-5p in EVs released by ovarian cancer cell lines increased fibronectin and vitronectin expression in peritoneal mesothelial cells, promoting cancer cell invasion [90].

### 2.4. Reprogramming Energy Metabolism

Tumor cells can reprogram their energy metabolism to fuel their rapid cell growth and proliferation. The role of tumor-derived EVs on this mechanism has also been shown. For example, our group demonstrated that EVs derived from leukaemia or lung cancer multidrug resistant cells induced a metabolic switch in recipient drug-sensitive cancer cells, by causing a decrease in the pentose phosphate pathway and an increase in glycolysis [128]. Other authors found that EVs from adriamycin-resistant breast cancer cells contained high amounts of glutathione S-transferase P1 (GSTP1, a phase II metabolic enzyme capable of detoxifying damaging chemicals from cells), which was transferred to sensitive cells. This study also showed that EVs isolated from the serum of breast cancer patients who did not respond to chemotherapy had higher GSTP1 levels than EVs from patients who responded to the treatment [91]. Interestingly, EVs secreted by breast cancer cells reprogrammed glucose metabolism in recipient non-tumor cells through the transfer of miR-122, which facilitated disease progression. Moreover, orthotopic mammary xenografts injected with EVs containing high levels of miR-122 presented reduced uptake of glucose into the brain and lungs, giving rise to a reduced expression of pyruvate kinase and glucose transporter 1, GLUT1 [92]. 

### 2.5. Transferring Mutations

Evidence suggests that tumor-derived EVs contain pieces of DNA that might comprise the entire genome. It is still not understood how DNA is packaged inside EVs and if DNA transferred by EVs is functional in recipient cells [129]. However, recent literature suggests some functional role for DNA transferred by EVs. Indeed, the fusion genes PTPRZ1-Met can be present on the cargo of EVs released by glioblastoma cell lines and transferred to other glioblastoma cells, causing a more aggressive phenotype in the recipient cells. In vivo, xenografts injected with EVs carrying PTPRZ1-Met harbour larger tumors, confirming the role of those EVs containing PTPRZ1-Met in tumorigenesis [93]. The truncated and functional forms of Alk (Anaplastic Lymphoma Kinase) mRNAs were also found in the cargo of EVs released by melanoma drug resistant cells, being transferred to sensitive cells and activating the MAPK signaling pathway in the recipient cells [95]. Similarly, aggressive glioma cells expressing EGFRvIII, a truncated oncogenic form of the epidermal growth factor receptor (EGFR), released EVs containing EGFRvIII which were taken up by recipient glioma cells lacking this isoform, thus promoting the activation of the MAPK and Akt signaling pathways [94]. EVs derived from colorectal cancer cells contained the oncogenic mutant β-catenin which activated WNT signaling in recipient cells with wild-type β-catenin, promoting cancer progression [96]. In addition, EVs released by epithelial ovarian cancer cell lines carrying SMAD4 mutations enhanced platinum-resistant phenotype in recipient drug-sensitive ovarian cancer cells, suggesting a possible transfer of SMAD4 mutations through EVs [97]. 

### 2.6. Modulating the TME: Evading Immune Response and Promoting Inflammation

Most tumor cells express antigens that can mediate recognition by the immune system. However, some cancer cells are able to evade the antitumor immune response and continue proliferating. Also, tumor progression is closely related to chronic inflammatory processes and may involve deregulation in activity of various types of immune cells. Remarkably, several studies have extensively described the role of EVs released by tumor cells on different types of neighbouring cells present in the surrounding TME and which are involved in inflammation or immune response. 

Most of the studies revealed the impact of EVs on TME modulation towards a tumor promoting and supporting environment. Nevertheless, we should be aware that some reports have shown contradicting data, with EVs inducing an anticancer immune response. For example, EVs derived from heat-stressed tumor cells converted immunosuppressive regulatory T cells into T helper cells, which contributed to their potent antitumor effect [130]. Also, EVs from leukemia cells injected into a mouse tumor model prevented tumor formation [131].

#### 2.6.1. Impact of Tumor-Derived EVs on Macrophages

Many studies show that EVs released by tumor cells can transfer their cargo to macrophages. For example, EVs released by ovarian cancer cells transferred the oncogenic miR-1246 to M2-type tumor-associated macrophages but not to M0-type naïve macrophages, suggesting that EVs from cancer cells interfere with the TME, which then plays a role in tumor progression [98]. Indeed, miR-1246 was also detected on EVs released by colon cancer cells and could reprogram macrophages to induce the production of tumor supportive factors, such as IL-10 and metalloproteinases (MMPs) [100]. Furthermore, Casadei et al. demonstrated in vitro that miR-25-3p and miR-921-3p present in EVs secreted by liposarcoma cell lines were capable of stimulating the secretion of the pro-inflammatory cytokine IL-6 from macrophages, which in turn stimulated cancer cell proliferation. In addition, the presence of those miRNAs in EVs isolated from the plasma of liposarcoma patients was confirmed [103]. Another study reported that EVs isolated from glioblastoma cells containing high levels of miRNAs (such as miR-21) were taken up by macrophages in a glioma-bearing brain mouse model, confirming the influence of EVs released by glioblastoma cells in the TME [104]. Also, EVs released by hepatocellular carcinoma cell lines contain the lncRNA TUC339 which can regulate macrophage activation and induce an M2 macrophage polarization, favoring cancer cell progression [106]. Interestingly, EVs released by ovarian cancer cell lines under hypoxia conditions contained high levels of miR-940, inducing macrophage M2 polarization [132]. However, contradictory data showed that EVs shed by melanoma cell lines activated macrophages, and also helped the maturation of dendritic cells and enhanced T-cell proliferation. Nonetheless, this study showed mixed biological effects, with the role of EVs not only in promoting antitumor immune response, but also in tumor immune escape [133].

#### 2.6.2. Impact of Tumor-Derived EVs on T Lymphocytes

The T lymphocytes may also be modulated by tumor-derived EVs. For example, some EVs derived from glioblastoma cells expressed the transmembrane protein PD-L1, which has the potential to bind PD1 and block T-cell activation and proliferation [105]. Furthermore, EVs shed by nasopharyngeal carcinoma cell lines promoted T-cell dysfunction, which was mediated by miR-24-3p through repression of targeting fibroblast growth factor (FGF)11 [108]. Moreover, the miR-24-3p was markedly enriched in EVs derived from nasopharyngeal carcinoma patients’ serum when compared to healthy donors and correlated with worse disease-free survival of patients [109]. Other studies have also demonstrated that colon cancer cell lines released EVs containing members of the carcinoembryonic antigen related cell adhesion molecule (CEACAM)-family. These influenced T-cell behavior [101] or induced phenotypic alterations on T-cells via activation of the TGF-β/Smad signaling [134], supporting tumor cell growth. In addition, EVs released by breast cancer cells suppressed T-cells by delivering TGF-β directly to these immune cells [135]. EVs shed by kidney adenocarcinoma cells triggered T-cell apoptosis through the transfer of the Fas ligand [111]. Similarly, other cancer types such as melanoma, prostate, oral and colorectal were shown to release EVs containing the Fas ligand which were taken up by T-cells [136,137,138,139], again demonstrating the significant role of EVs in eliminating the most effective cells with antitumor response. Interestingly, like the Fas ligand, galectin-9 also mediates apoptosis when bound to its receptor. EVs derived from nasopharyngeal carcinoma cells (which contained galectin-9) were taken up by mature T cells. The interaction between galectin-9 and the Tim-3 receptor from T cells triggered T cells apoptosis [110]. Moreover, EVs released by nasopharyngeal carcinoma cells liberated miRNAs which through inhibition of the MAP-kinase pathway, originated a decrease in T-cell proliferation [109]. Furthermore, EVs derived from ovarian carcinoma cells contained the metabolic checkpoint molecule arginase-1, which suppressed T-cell responses and promoted tumor growth [99]. Curiously, although to a large extent unexplored, the modulation of B-lymphocytes by EVs has also been reported. EVs shed by esophageal cancer cell lines induced naïve B cells differentiation into TGF-β-producing regulatory B cells, contributing to immune suppressor functions on T-cell proliferation [140]. 

#### 2.6.3. Impact of Tumor-Derived EVs on Fibroblasts

EVs-mediated communication between tumor cells and fibroblasts has been reported. An in vitro study showed that EVs shed by chronic lymphocytic leukemia cells were actively incorporated by surrounding stromal cells, inducing features of cancer-associated fibroblasts (CAFs), resulting in the secretion of inflammatory cytokines that contributed to a tumor-supportive microenvironment. This effect was also observed in vivo, by injecting labeled EVs into mice and following the targeted cells [141]. In addition, EVs released by ovarian cancer cell lines [142] or by Hodgkin lymphoma cells [143] modulated normal fibroblasts behavior, altering their phenotype (activating them to a CAFs-like state) to support tumor growth and progression. Interestingly, EVs released by gastric cancer cell lines enhanced pericytes proliferation and migration, and induced the expression of CAFs markers in pericytes [144].

The function of miRNAs delivered via EVs as messengers between tumor cells and fibroblasts at the TME has also been described. For example, the miR-21 present in EVs from hepatocellular carcinoma cell lines aided tumor progression by converting normal hepatocytes stellate cells to CAFs. Clinical data also indicated that high levels of EVs containing miR-21, isolated from the serum of hepatocellular carcinoma patients, were correlated with a greater activation of CAFs and higher vessel density [107]. Interestingly, the miR-675 present in EVs released by metastatic osteosarcoma cell lines down-regulated CALN1 expression on non-malignant fibroblast cells, increasing their migration and invasion capacities [112]. Similarly, primary pancreatic fibroblasts isolated from mice were converted into CAFs-like cells in the presence of EVs released by pancreatic cancer cell lines, in a process mediated by miR-155 contained in the EVs cargo [145]. Moreover, miRNAs present in EVs released by several tumor cells induced in vitro the reprogramming of fibroblasts into CAFs, to support tumor growth. This was shown in EVs derived from gastric cancer, colorectal cancer, melanoma and lung cancer cells and was mediated by the transfer of miR-27a [113], miR-10b [102], miR-155-5p [114] and miR142-3p [115], respectively.

## 3. Tumor-Derived EVs Affecting Cancer Therapy Resistance

**Figure 3:**
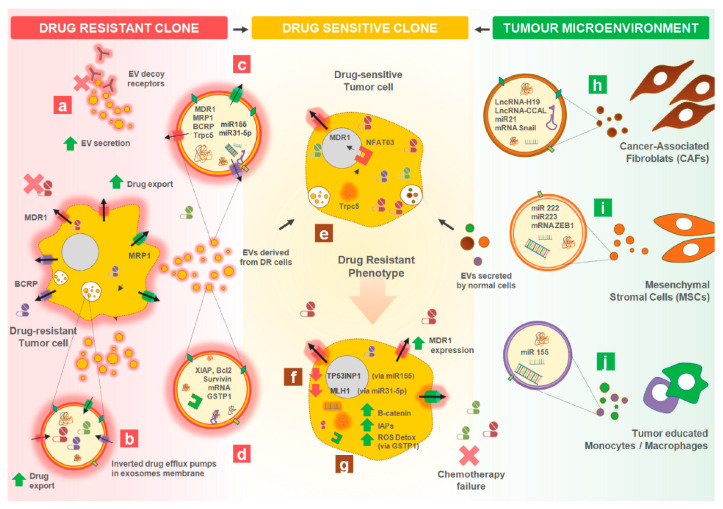
Impact of tumor-derived EVs in Cancer Therapy Resistance. EVs secreted from the few drug resistant (DR) cancer cells (on the left) or by normal cells within the tumor microenvironment (on the right) can contribute to exacerbate resistance in otherwise drug sensitive cancer cells (centre). (**a**) Exacerbated production of EVs by drug resistant cancer cell clones carrying on their surface the same tumour markers. These EVs will be targeted by certain monoclonal antibody-based therapeutics (e.g., trastuzumab, rituximab, etc.) and act as decoy receptors, lowering the availability of these targeted therapies to cancer cells. Additionally, in DR EVs (**b**) the drug efflux pumps may be inverted in the membrane of exosomes due to their biogenesis, when compared to their normal orientation in their donor cells. This can promote cytoplasmatic drug-influx into those EVs that will act as a drug efflux delivery system. EVs from a minor fraction of cancer drug resistant clones will (**c**) transfer functional drug efflux pumps (e.g., MDR1, MRP1, BCRP) to drug sensitive recipient cancer cells. This will enable recipient cells to efflux the drugs reducing the intracellular drug concentrations to sublethal levels. DR cancer cells can (**d**) promote the induction of anti-apoptotic pathways in recipient drug sensitive cells by transferring anti-apoptotic proteins (e.g., XIAPs, Bcl2, IAPs, Survivin, etc.). (**e**) Drug resistant clones also transfer TrpC5 protein through EVs to drug sensitive counterparts, activating the transcription factor NFATc3 and MDR1 gene expression. This will originate MDR1 efflux pumps production in recipient cells. (**f**) DR cell-derived EVs carry miR-31-5p that when internalized by drug sensitive counterparts promote down-regulation of MLH1 and consequently inhibit the mismatch repair system. This can lead to increased genomic instability and an aggressive phenotype in recipient cells. (**g**) DR cell-derived EVs have increased levels of GSTP1 mRNA/proteins that improve reactive oxygen species (ROS) detoxification in drug sensitive cells. Within the tumour microenvironment, stromal cells secrete EVs that will support a drug resistance phenotype in otherwise drug sensitive cancer cells. Indeed, (**h**) EVs from Cancer-associated Fibroblasts (CAFs) carry lncRNAs that will activate the β-catenin pathway in recipient cells inducing a cancer stem cell-like phenotype. Similarly, (**i**) EVs shed from Mesenchymal Stromal Cells (MSCs) have in their cargo miR-222/miR-223 and ZEB1 mRNA or (**j**) EVs secreted by monocytes carry miR155 that enable cancer resistance to several drugs (e.g., gemcitabine and cisplatin).

Extracellular vesicles may promote resistance to chemotherapy and targeted therapy in cancer, including multidrug resistance (MDR). This is due to the intercellular transfer of drug resistant traits between cancer cells or between cancer cells and the tumor microenvironment, as explained in the following sections (Figure 3).

### 3.1. Intercellular Transfer of Drug Resistant Traits between Cancer Cells

The mediators of the intercellular transfer of drug resistant traits between resistant and sensitive cancer cells will be discussed in the following sections (Table 2).

#### 3.1.1. Transferring Drug-Efflux Pumps

EVs shed by drug resistant cancer cells may transfer drug efflux pumps to sensitive recipient cancer cells, causing drug efflux in the recipient cells, thereby reducing drug concentrations to sublethal levels [3,196,197]. Amongst the drug efflux pumps, various members of the ATP-binding cassette (ABC) family, namely breast cancer resistance protein (BRCP/ABCG2), ABCA3, multidrug resistance-associated protein 1 (MRP1/ABCC1), and P-glycoprotein (P-gp/MDR1/ABCB1) [146,147,157,198,199] have been shown to be transferred by EVs from drug-resistant cells to drug-sensitive cells. The orientation of these transporters may be inverted in some EVs when compared to their orientation in their donor cells, thus possibly promoting drug-influx rather than efflux into EVs [197].

The non-genetic acquisition of P-gp mediating drug resistance or enrichment on this drug efflux pump in EVs shed by drug-resistant cells has been reported in many studies. These include castration-resistant prostate cancer cells after docetaxel exposure [148,149], breast cancer cells following docetaxel [150], adriamycin [151,152,153], or doxorubicin [154] exposure in paclitaxel-resistant ovarian cancer cells [156], doxorubicin-resistant osteosarcoma cells [155], in multidrug-resistant leukemia [146,157], and in neuroblastoma cells [158]. Intercellular transfer of functional P-gp has also been reported in vivo in a neuroblastoma xenograft mouse model [158] and breast cancer xenograft model [200].

It is unclear whether the intercellular transfer of the EVs cargo is a selective or a random process. The transfer of P-gp through EVs released by drug-resistant leukemia cells displayed no cell-type selectivity between breast and lung cancer recipient cells [163]. Additionally, this cargo was reported to be transferred (along with MRP1) to both malignant and non-malignant recipient cells whereas EVs from breast cancer cells only transferred P-gp to malignant cells [200].

It was recently reported the EV-mediated intercellular transfer of ABCB1 protein from drug-resistant KBv200 cells to drug-sensitive KB cells constituted one mechanism behind the acquisition of a resistant phenotype, following exposure to either vincristine, cisplatin or doxorubicin [159]. Interestingly, as the tested chemotherapeutic agents possess distinct chemical structures and mechanisms of action, the authors concluded that independently of being an ABCB1 substrate, conventional chemotherapeutic drugs can still promote the transfer of this protein through EVs [159]. Another study reported that MRP1-containing EVs were responsible for the transfer of a multidrug resistance phenotype from resistant HL-60 cells to recipient cells, and highlighted the differential expression of miR-19b and miR-20a between EVs from chemo-resistant and chemo-sensitive cells [146].

Moreover, human acute lymphoblastic leukemia cells (CCRF–CEM) exposed to EVs from their multidrug-resistant subline (VLB_100_) displayed increased expression of P-gp. This could not be explained by the transcription and posterior translation of the MDR1 gene (due to the short incubation time of two and four hours), suggesting that there was a direct intercellular transfer of this drug efflux pump [157]. 

Moreover, a prolonged expression of P-gp that extended well beyond the half-life of this protein was reported after a single exposure of a breast tumor (MCF7) xenograft model to EVs retrieved from drug-resistant cells [200]. This demonstrates the EVs’ capacity to disseminate a stable resistant phenotype and suggests the concomitant action of the transferred P-gp with other transferred cargo such as miRNAs, mRNAs or possibly DNA fragments [3,14]. Indeed, it has been demonstrated that the knock-down of the MDR1 gene in donor cells does not impede the transfer of resistance, suggesting that other mechanisms are involved in this process [201].

Adriamycin-resistant MCF7 cells transfer TrpC5 protein through EVs to recipient cells, activating the transcription factor NFATc3 which in turn leads to MDR1 gene expression, culminating in P-gp production in recipient cells [152]. Of interest is a study with breast cancer patients reporting TrpC5-containing EVs in only those who received chemotherapy [202]. 

#### 3.1.2. Transferring Apoptotic Modulators

Other studies report EV-mediated drug resistance through the targeting of apoptosis regulators, promoting the induction of anti-apoptotic pathways in recipient cells such as the Bcl-2/BAX signaling [185]. Kreger et al. reported that an enrichment in the anti-apoptotic protein survivin in EVs retrieved from MDA-MB-231 triple-negative breast cancer cells previously treated with paclitaxel, induced resistance to this chemotherapeutic drug and promoted survival of serum-starved or paclitaxel-treated fibroblasts and SKBR3 breast cancer cells, when co-incubated with the same EVs [162]. Moreover, EVs from drug-resistant chronic myeloid leukemia cells gave rise to an accumulation in recipient cells not only of P-gp and microRNAs (miR-27a, miR-451 and miR-21) related to P-gp expression, but also of the inhibitors of apoptosis proteins XIAP, IAP and survivin. These EVs induced drug resistance in drug-sensitive recipient breast and lung tumor cells, thus suggesting multifactorial mechanisms for drug resistance and apoptosis evasion [163].

#### 3.1.3. Transferring Other Proteins

A recently published paper on proteasome variations of EVs retrieved from two cisplatin-resistant and one sensitive oral squamous cell carcinoma cell lines, reported 77 differentially expressed proteins in EVs from both resistant cell lines, predominantly downregulated and involved in EGFR-associated networks [164]. Interestingly, six of these proteins were involved in the regulation of metal ion transportation, (e.g., ATP1A1 and ATP1B3) and since cisplatin is a metal-based compound, their down-regulation may have halted the uptake of this drug by the resistant cells [164]. Of the four found upregulated proteins, TGM2 had previously been reported to play a role in EV selective packaging of cargo and in mediating chemoresistance in lymphoma and breast cancer [164,203,204]. Moreover, a recent proteomic study reported the enrichment of GSTP1 and p-STAT3 in EVs from 5-fluorouracil-resistant colorectal cancer cells and the role of p-STAT3 in the transfer of resistance was further confirmed in vivo [165]. Furthermore, Zhao et al. recently postulated that the EV-mediated transfer of CLIC1 protein induced vincristine resistance in gastric cancer cells in vitro, an effect suggested to be related to the up-regulation of P-gp and Bcl-2 [166].

#### 3.1.4. Transferring microRNAs, mRNAs and lncRNAs

Other molecules in the EV cargo which may be involved in the intercellular transfer of drug resistance include microRNAs, functional mRNAs and lncRNAs as well as regulators of these molecules and the above-mentioned molecules (in Section 3.1.1, Section 3.1.2, Section 3.1.3) [3,186,187,205,206]. Some studies suggest that, in addition to the direct transfer through EVs of known inducers of multidrug resistance, an EV-mediated increase in the expression of these inducers in recipient cells may also occur. 

##### miRNAs

Some studies have attempted to associate specific EV-transferred microRNAs to drug resistance. EVs isolated and purified from a multidrug-resistant chronic myeloid leukemia cell line induced drug resistance in MCF7 cells after co-culture, through the EV-mediated transfer of miR-21 which possibly led to the activation of the Akt signaling, thus regulating the NF-κB pathway [163]. Moreover, in lung cancer cells, cisplatin-resistance was mediated by EV’s miR-96 which inhibited LIM-domain only protein 7 expression [168] whereas in breast cancer the miR-222 from EVs contributed to adriamycin-resistance [169]. Indeed, EVs from triple-negative breast cancer cells induced significantly higher docetaxel and doxorubicin resistance on non-tumor breast cells and led to differential expression of 138 genes and 70 miRNAs, where downstream genes of the MAPK pathway, such as R-RAS and MAPK3, as well as miR-155-5p, miR-542-3p, let-7, and miR-28, might have played important roles as mediators [170]. Another study compared the microRNA content of EVs isolated from three drug-resistant ovarian cancer cell lines (taxol-resistant SKOV3, cisplatin-resistant A2780 and multidrug-resistant HEYA8) versus their drug-sensitive counterparts and detected the presence of miR-183-5p in all three resistant cell lines. This study also suggested that miR-183-5p possibly induced drug-resistance by modulating MECP2, thus regulating cell proliferation and influencing the biological process of response to hypoxia [171]. Moreover, in pancreatic cancer, EVs’ miR-155 mediated gemcitabine-resistance through the targeting of the pro-apoptotic p53 target gene TP53INP1 [172], repression of the gemcitabine-metabolizing gene DCK and promotion of ROS detoxification by transferring SOD2 and CAT transcripts [173]. In addition, the intercellular transfer of miR221/222 mediated by EVs promoted tamoxifen-resistance in ER-positive breast cancer MCF7 cells, through p27 and estrogen receptor α downregulation [174]. Another study showed that exosomal miR-19b promoted oxaliplatin-resistance in SW480 colorectal cancer cells and that the inhibition of this microRNA led to increased drug sensitivity [175]. Interestingly, the secretion of EVs containing tumor-suppressor miR-145 and miR-34a correlated positively with 5-fluorouracil resistance in human colon cancer DLD-1 cells [176]. Furthermore, EVs from sorafenib-resistant renal cell carcinoma cell lines promoted resistance to this drug in vitro and in vivo, through the transfer of miR-31-5p which directly promoted down-regulation of MLH1 expression, one of the seven proteins that constitute the mismatch repair system [177]. Additionally, elevated levels of miR-31-5p were found in circulating EVs from renal cell carcinoma patients with progressive disease during sorafenib therapy when compared to pre-therapy levels [177]. Other work demonstrated (by using microarray technology in four different lines of synovial sarcoma) that EV-encapsulated microRNA-761 enhanced pazopanib resistance, possibly through the downregulation of TRIP6, LMNA and SIRT3 expression [178]. Yin et al. also suggested that the EV-encapsulated miR-1238 plays a role in mediating temozolomide-resistance in glioblastoma cells in vitro and in vivo, by acting on CAV1 and activating the EGFR-PI3K-AKT-mTOR pathway [179]. Furthermore, a recent study using high throughput technology has reported the upregulation of two pseudogenes (a novel pseudogene and RNA 5.8S ribosomal pseudogene 2) in EVs released by both non-small cell lung cancer (NSCLC) and chronic myeloid leukemia MDR tumor models. The same study also observed increased levels of a miRNA panel (miR-204-5p, miR-139-5p, miR-29c-5p, miR-551b-3p, miR-29b-2-5p, and miR-204-3p) in the lung cancer model, when compared to their drug-sensitive counterparts [207]. Moreover, in NSCLC, cisplatin-resistance was induced by the transfer of miR-425-3p by EVs shed by resistant cells, which facilitated autophagic activation by targeting AKT1 in recipient cells [180,181]. Another recent study reported that miR-744 (downregulated in EVs retrieved from sorafenib-resistant hepatocellular carcinoma cells and patient serum) can inhibit proliferation and sorafenib chemoresistance in HepG2 cells by targeting PAX2, thus being suggested as a molecular target for the development of a possible innovative treatment strategy [182]. Similarly, the downregulation of miR-100-5p in EVs retrieved from cisplatin-resistance lung cancer cells was capable of inducing resistance in recipient cells, an effect further confirmed in vivo [183]. Work using multidrug resistant leukemia cells showed that EVs transferred ABCB1 transcript to recipient cells, which suppressed the transporter ABCC1 through miR-326 [199]. 

##### mRNAs

Some mRNAs are responsible for the intercellular transfer of drug resistance. Using an ovarian cancer xenograft mouse model, DNA methyltranferase 1 (DNMT1) mRNA was shown to play an important role in EV-mediated cisplatin-resistance [184]. Moreover, the transfer of EVs from adriamycin-resistant breast cancer cells (which had increased levels in GSTP1 mRNA - coding for a drug-detoxifying enzyme) increased GSTP1 expression and induced resistance in recipient cells in an EV concentration-dependent manner [91].

##### lncRNAs

The lncRNAs linc-ROR [186] and linc-VLDLR [187], transferred by EVs, induced sorafenib and doxorubicin-resistance in hepatocellular carcinoma HepG2 cells, through induction of TGFβ and increased expression of ABCG2, respectively. The EVs-Linc-VLDLR-ABCG2 pathway also plays a role in promoting multidrug resistance in esophageal cancer cells [188]. Additionally, EV-mediated intercellular transfer of lncARSR (which promoted Sunitinib resistance through competitive binding with miR-34/miR-449) increased AXL and c-MET expression in renal cell carcinoma cells. Also, the levels of lncARSR found in the plasma and tumor tissues correlated with Sunitinib response in renal cell carcinoma patients [189]. Interestingly, the transfer of lncRNA HOTTIP through EVs promoted cisplatin resistance in gastric cancer cells by activating the HMGA I/miR-218 Axis. Moreover, high levels of EVs’ lncRNA HOTTIP in patient serum also correlated with poor response to cisplatin treatment [190]. Of interest is that the EV-shuttled lncRNA HNF1A-AS1 acted as a competing endogenous RNA of miR-34b, upregulating TUFT1 and inducing cisplatin-resistance in cervical cancer cells [191]. Moreover, the spread of temozolomide-resistance by EVs from glioblastoma cells containing lncRNA SBF2-AS1 was confirmed both in vitro and in vivo, and its effect was suggested to be mediated by competition with miR-151a-3p to disinhibit XRCC4, a protein responsible for double-strand repair [192]. Finally, the lncRNA AGAP2-AS1 packaged into EVs, was found to promote trastuzumab resistance in two HER-2 positive breast cancer cell lines [193].

#### 3.1.5. Transferring Lipids

Lipids have been shown to participate in EV-mediated drug resistance. For example, ceramide, which plays a role in EV biogenesis and cargo loading, also mediates drug resistance possibly through P-gp [208,209,210]. Additionally, a lipidomic study revealed variations in the expression of 35 phospholipids between EVs retrieved from a gefitinib-resistant lung cancer cell line and its sensitive parental cell line [194]. Moreover, acid sphingomyelinase expression in EVs from multiple myeloma cell lines increased following exposure to melphalan and bortezomib, leading to the transfer of a drug-resistant phenotype to chemosensitive cells. In addition, acid sphingomyelinase inhibition by amitriptyline resulted in increased drug sensitivity in recipient multiple myeloma cells and in primary multiple myeloma cells [195]. 

### 3.2. Intercellular Transfer of Traits between the Microenvironment and Tumor Cells

The bidirectional crosstalk between the TME and tumor cells also plays a fundamental part in EV-mediated drug resistance. Within stromal cells, EVs from CAFs induced: (i) resistance to 5-fluorouracil and oxaliplatin in colorectal cancer stem cells either retrieved from patient-derived mice xenografts or by sorting for CD133+ in colorectal cancer cells lines [211]; (ii) chemoresistance in colorectal cancer cells in vitro and in vivo through transfer of exosomal lncRNA H19 that activates the β-catenin pathway [212]; (iii) resistance to gemcitabine in pancreatic adenocarcinoma cells via the transfer of both mRNA encoding the resistance factor Snail and its target miR-146a [213]; (iv) oxaliplatin resistance in colorectal cancer cells through the transfer of the colorectal cancer-associated lncRNA (CCAL) and activation of the β-catenin pathway both in vitro and in vivo [214]; (v) cisplatin-resistance in head and neck cancer cells through the transfer of miR-196a, which targets CDKN1B and ING5 [215], and (vi) alongside cancer associated adipocytes, also lead to ovarian cancer cell resistance to paclitaxel through the transfer of miR-21, which binds to the APAF1 coding sequence and downregulates APAF1 expression [216].

Stromal cells EVs induced resistance in acute lymphoblastic leukemia cells through the transfer of galectin-3, leading to the NF-kB pathway activation [217]. These EVs also induced resistance to bortezomib in multiple myeloma cells, possibly through the activation of JNK, p38, p53, and Akt [218], and in breast cancer by stimulating NOTCH3 and the pattern recognition receptor RIG-I, which in turn activates a STAT1-dependent antiviral signaling [219].

Mesenchymal stem cells caused drug resistance in recipient breast cancer cells by releasing EVs with miR-222/miR-223 [220]. Additionally, ZEB1 mRNA, encapsulated into EVs from a mesenchymal NSCLC line, transferred gemcitabine and cisplatin resistance to a surrounding epithelial NSCLC line [221].

Fascinatingly, miR-21 containing EVs from neuroblastoma cells induced the release of monocyte EVs containing miR-155 which in turn induced cisplatin resistance on neuroblastoma cells, by entering these cells and repressing TERF1, both in vitro and in vivo [222].

### 3.3. Drug Efflux Mediated by EVs

Drug efflux from cancer cells is made possible through the secretion of drugs into the cargo of EVs shed by those cells [3,197,223]. Indeed, it has been suggested that: (a) cancer cells secreting more EVs achieve the greatest levels of resistance [149,164,223,224,225,226] and that (b) drug-resistant cells can export larger quantities of drugs into their EVs than drug-sensitive cells [164,167,197,225]. Interestingly, a massive increase in the release of EVs has been reported to be one of the responses of cancer cells to photodynamic treatment and chemotherapeutic drugs, both in vitro and in vivo [227].

### 3.4. Increased Release of EVs by Drug Resistant Cells

Some studies suggest that drug resistant cells produce more EVs than drug sensitive cells [147,149,197,228,229,230]. Additionally, many studies report a direct association between the presence of some mediators of drug resistance and molecules involved in EV production, supporting the findings that drug resistant cells release more EVs than sensitive cells. For example, Annexin A3 that is involved in ovarian cancer cellular resistance to platinum [230,231] and is detected in EVs from the same type of cancer resistant to cisplatin, also appears to be responsible for an increased production of EVs in the same cells [230]. Moreover, ABCG2 present in EVs has not only been reported to mediate resistance to mitoxantrone [160], topotecan, imidazoacridinones and methotrexate [161] in breast cancer cell lines, but has also been found to exert a role in EV production [229]. Interestingly, ABCA3 is not only a drug-efflux pump but also a modulator of EV-release from B-cell lymphoma cells; furthermore, these exosomes shielded target cells from rituximab [147]. Similarly, trastuzumab was inefficient at inhibiting breast cancer cell proliferation when in the presence of HER2-carrying EVs [232]. Moreover, the activation of this receptor (which dimerizes with EGFR or HER3) stimulates EV production [232]. Curiously, the transfer of lncRNA small nucleolar RNA host gene 14 (SNHG14) by EVs shed by trastruzumab-resistant HER2+ breast cancer cells has also been reported to be a resistance mechanism to this antibody [185]. A comprehensive study in pancreatic ductal adenocarcinoma cell lines reported that an increase of miR-155 expression levels in cells transfected with pre-miR-155 caused an increase in the secretion of EVs and an increase in miR-155 expression levels on the released EVs content. Those EVs delivered miR-155 into other pancreatic ductal adenocarcinoma cancer cells, inducing gemcitabine-resistance in recipient cells [172].

A recent review has highlighted the role of RAB7A protein in cancer progression, EV secretion and EV-mediated cisplatin resistance in ovarian and cervical cancer cells [233]. The authors previously demonstrated that the downregulation of RAB7A increased cisplatin resistance in cervical cancer cell lines, which correlated with the increased production of EVs and reduction of cisplatin intracellular concentration, suggesting that chemoresistance resulted from a greater export of cisplatin through EVs [167]. Dorayappan et al. also reported that hypoxia increased the release of exosomes in ovarian cancer cells (through the activation of STAT3, up-regulation of RAB27A and down-regulation of RAB7, LAMP1/2 and NEU-1) and also increased the EV-mediated efflux of cisplatin from these cells [224].

## 4. Conclusions

The vast majority of experimental evidence reviewed herein suggests a key role for EVs in favouring cancer hallmark traits throughout the distinct stages of cancer progression. Most importantly, we demonstrate that this cancer EVs signaling will ultimately favour the emergence of drug resistance. Thus, EVs are instrumental for the survival of resistant cancer clones which constitute a reservoir of minimal residual disease, responsible for the post-therapy refractory relapses in all human cancers. 

Despite the scientific robustness, most experimental evidence on EVs was obtained from in vitro experiments and to a lesser extent in vivo models, thereby hampering the in-depth understanding of the cancer EVs signaling network with respect to clinical application. Although signifying an important proof-of-concept, these studies also seem to indicate that the EVs selective packaging and cargo is a highly dynamic process that relies on the experimental conditions and type of drug exposure. Thus, further studies in physiologically relevant models that recapitulate the TME throughout different stages of cancer progression will be a perquisite towards a more profound understanding of the tumor promoting EV signaling network. Unfortunately, most clinical research studies on EVs were based on very small sample sizes and require further validation in large clinical trials. This may justify in part why only few EV-based technologies are currently available for clinical cancer diagnosis/prognosis. It is now almost certain that EVs profiling could provide clinical guidance to predict tumor progression and unravel new strategies to regain control of disrupted EVs signaling network to our favour, either as EV-based drug delivery or as tumor EVs depletion strategies.

Taken together, a growing body of preclinical and clinical evidence reveals that EVs hold great potential as diagnostic cancer biomarkers or as naturally engineered carriers for targeted drug delivery, with some of these EV-based technologies being capable of reaching the market. Nevertheless, the main scientific challenges, including the development of highly sensitive single tumor-EV detection tools and the understanding of tumor EV trafficking and uptake, still need to be addressed to fully translate EV research into clinically reliable tools for cancer therapeutic applications. 

## Figures and Tables

**Table 1:** EVs cargo derived from different types of cancers with impact in the tumor hallmarks.

Cancer Type from Released EVs	EVs Cargo	Type of Study	Refs
**Promoting cell proliferation and escape from apoptosis**			
**Glioblastoma**	Splicing factor RBM11; CLIC1	In vitro, In vivo	[48,49]
**Melanoma**	PDGFR-β	In vitro	[50]
**Pancreatic cancer**	Zinc transporter ZIP4; miR-23b-3p; miR-222	In vitro, In vivo, Patients samples	[51,52,53]
**Breast cancer**	miR-1246	In vitro	[54]
**Cholangiocarcinoma**	miR-205	In vitro	[55]
**Colon cancer**	DNp73 mRNA; miR-193a; mir-200b; lnRNA PVT1	In vitro, In vivo	[56,57,58,59]
**Thyroid cancer**	miR-222; miR-146b	In vitro	[60]
**Ovarian serous carcinoma**	miR-21	In vivo, Patients samples	[61]
**Acute leukemia**	miR-118, miR-116	In vivo, Patients samples	[62]
**Gastric cancer**	lnRNA ZFAS1	In vitro	[63]
**Esophageal cancer**	miR-93-5p; lncRNA ZEB1-AS1	In vitro, Patients samples	[52,64]
**Hepatocelular carcinoma**	lncRNA TUC339	In vitro	[65]
**Sustaining angiogenesis**			
**Glioblastoma**	lnRNA CCAT2; lncRNA POU3F3; miR-21; CXCR4 receptor; VEGF	In vitro, In vivo	[66,67,68,69]
**Head and neck squamous cell carcinoma**	EPHB2	In vitro, In vivo	[70]
**Hepatocellular carcinoma**	Vasorin; miR-103	In vitro, In vivo	[71,72]
**Multiple myeloma**	piRNA-823	In vivo	[73]
**Colon cancer**	miR-25-3p	In vitro, In vivo	[74]
**Lung cancer**	miR-14-3p; miR-145-5p; miR-23a	In vitro	[75,76]
**Ovarian carcinoma**	miRNA-141-3p	In vitro	[77]
**Oral cancer**	miR-142-3p	In vitro, In vivo	[78]
**Nasopharyngeal carcinoma**	miR-23a	In vitro, Patients samples	[79]
**Contributing to cancer cell invasion and metastasis**			
**Cholangiocarcinoma**	miR-205-5p	In vitro	[55]
**Breast cancer**	Caveolin-1	In vitro	[80]
**Colon cancer**	Wnt5b; AREG	In vitro	[81,82]
**Hepatocarcinoma**	CXCR4; SMAD3; miR-93; miR-103	In Vitro; In vivo; Patient Samples	[72,83,84,85,86]
**Gastric cancer**	miR-423-5p	In vitro, In vivo; Patient samples	[87]
**Prostate cancer**	miR-1246	In vitro, In vivo; Patient samples	[88]
**Glioblastoma**	miR-148a	In vitro; Patient Samples	[89]
**Ovarian Cancer**	miR-99a-5p	In vitro	[90]
**Reprogramming energy metabolism**			
**Breast Cancer**	GSTP1; miR-122	In vitro; In vivo; Patient samples	[91,92]
**Transferring mutations**			
**Glioblastoma**	fusion genes PTPRZ1-Met, EGFRvIII	In vitro; In vivo	[93,94]
**Melanoma**	mRNA truncated Alk form	In vitro	[95]
**Colon cancer**	Mutant β-catenin	In vitro	[96]
**Ovarian cancer**	SMAD4	In vitro	[97]
**Modulating the Tumor Microenvironment: evading immune response and promoting inflammation**			
**Ovarian Cancer**	miR-1246, metabolic checkpoint molecular arginase-1	In vitro	[98,99]
**Colon cancer**	miR-1246; miR-10b; CEACAM-family; Fas ligand	In vitro	[100,101,102]
**Liposarcoma**	miR-25-3p; miR-921-3p	In vitro, In vivo, Patients samples	[103]
**Glioblastoma**	miR-21, PD-L1	In vitro	[104,105]
**Hepatocellular carcinoma**	lncRNA TUC339; miR-21	In vitro	[106,107]
**Nasopharyngeal carcinoma**	miR-24-3p, galectin-9	In vivo, Patients Samples	[108,109,110]
**Renal cell carcinoma**	Fas ligand	In vitro	[111]
**Osteosarcoma**	miR-675	In vitro	[112]
**Gastric Cancer**	miR-27a	In vivo; Patient samples	[113]
**Melanoma**	miR-155-5p	In vitro, In vivo	[114]
**Lung cancer**	miR142-3p	In vitro	[115]

**Table 2:** Intercellular transfer of drug resistant traits between resistant and sensitive cancer cells according to anti-cancer drug and cancer type.

Intercellular Transfer	Specific EVs Cargo Transferred	Anti-Cancer Drugs	Cancer Type	Cell Lines/ Model/ Patients Studies	Refs
**Drug-efflux pumps**	MRP1/ABCC1	Multi-drug	Leukemia,	HL-60	[146]
	ABCA3	rituximab	B-cell lymphoma	Su-DHL-4; Balm3; OCI-Ly1	[147]
	Pgp	Docetaxel Adriamycin Doxorubicin Paclitaxel Multi-drug	Prostate, Breast Breast Breast; Osteosarcoma Ovarian; Neuroblastoma; Leukemia	DU145 Tax-Sen/Tax-Res; 22Rv1 Doc-Res; MCF7 MCF7/ADM vs MCF7/WT MCF7;MG-63DXR30 A2780; BE (2)-C; VLB100; xenograft mouse models	[148,149,150] [151,152,153] [154,155] [156] [157,158]
	TrpC5	Adriamycin	Breast	MCF7	[152]
	ABCB1	Vincristine, cisplatin, doxorubicin	Oral squamous carcinoma	KBv200	[159]
	ABCG2	Mitoxantrone Topotecan	Breast	MCF7/MR; MCF7/FLV1000	[160]
		Imidazoacridinones methotrexate	Breast	MCF7/MR; MCF7/FLV1000	[161]
**Apoptotic modulators**	Survivin	Paclitaxel	Triple-negative Breast	MDAMB231	[162,163]
	Inhibitors of apoptosis proteins XIAP and IAP	Multi-drug	Breast Chronic myeloid leukemia Lungs	MCF7 CML562 A529	[163]
**Other Proteins**	ATP1A1ATP1B3	cisplatin	Squamous cell carcinoma	H314/H103	[164]
	TGM2	cisplatin	Squamous cell carcinoma	H314/H103	[164]
	GSTP1p-STAT3	5-fluoracil	Colorectal	RKO	[165]
	CLIC1	vincristine	Gastric	SGC-7901	[166]
	RAB7A	Cisplatin	Ovarian Cervical	HeLa; A431; 2008 (and cisplatin-resistant counterpart cell lines)	[167]
**miRNAs**	miR-21	Multi-drug	Chronic myeloid leukemia Breast	CMLK562 MCF7	[163]
	miR-96	Cisplatin	Lung	A549, H1299, MCF-7	[168]
	miR-222	Adriamycin	Breast		[169]
	miR-155-5p, miR-542-3p, let-7 and miR-28	Docetaxel Doxorubicin	Triple-negative Breast	MCF10A	[170]
	miR-183-5p	Taxol Cisplatin Multidrug	Ovarian	SKOV3, A2780, HEYA8	[171]
	miR-155	Gemcitabine	Pancreatic ductal adenocarcinoma	Panc1, MiaPaCa2, Colo-357 and PSN1 cell lines Patients samples, Murine xenograft model	[172,173]
	miR-221/222	Tamoxifen	Breast	MCF7	[174]
	miR-19b	Oxaliplatin	Colorectal	SW480	[175]
	miR-145 miR-34a	5-fluoracil	Colon cancer	DLD-1	[176]
	miR-31-5p	Sorafenib	Renal Cell Carcinoma	786-0, ACHN, Patients samples	[177]
	miR-761	Pazopanib	Synovial Sarcoma	SYO-1, HS-SYII, 1273/99, YaFuSS	[178]
	miR-1238	Temozolomide	Glioblastoma	U251 cell line, patient samples	[179]
	miR-425-3p	Cisplatin	Non-small cell lung cancer	A549 cell line, Patients samples	[180,181]
	miR-744	Sorafenin	Hepatocellular carcinoma	HepG2 cell line, Patients samples	[182]
	miR-100-5p	Cisplatin	Lung	A549	[183]
**mRNAs**	DNMT1 mRNA	Cisplatin	Ovarian cancer	Xenograft mouse model	[184]
	GSTP1 mRNA	Adriamycin	Breast	MCF7	[91]
**lncRNAs**	lncSNHG14	Trastruzumab	HER2-positive Breast	HER2-positive SKBR-3, HER2-positive BT474	[185]
	linc-ROR linc-VLDLR	Sorafenib Doxorubicin	Hepatocellular carcinoma	HepG2, Hep3B, PLC/PRF-5 and Huh-7	[186,187]
	linc-VLDLR-ABCG2	Multidrug	Esophageal cancer	Eca109 cell line, Patients samples	[188]
	lncARSR	Sunitinib	Renal Cell carcinoma	786-O, ACHN, xenograft mouse models	[189]
	lncHOTTIP	Cisplatin	Gastric	Xenograft mouse models, Patients samples	[190]
	lncHNF1A-AS1	Cisplatin	Cervical cancer	HeLa	[191]
	linc-SBF2-AS1	Temozolomide	Glioblastoma	U87, LN229, A172, T98, U251, Patients samples	[192]
	linc-AGAP2-AS1	Trastuzumab	HER2-positive Breast	HER2-positive SKBR-3, HER2-positive BT474	[193]
**Lipids**	Multiple phospholipids	Gefitinib	Lung	PC9R	[194]
	Acid Sphingomyelinase	Melphalan Bortezomib	Multiple myeloma	JJN3, LP1, OPM2, U266	[195]

## Abbreviations

ABC	ATP-binding cassette
*AGAP2*-*AS1*	AGAP2 Antisense RNA 1
ALIX	ALG-2-interacting protein X
Alk	Anaplastic Lymphoma Kinase
*APAF1*	Apoptotic Peptidase Activating Factor 1
AREG	amphiregulin
ARF6	ADP-ribosylation factor 6
*ATP1A1*	ATPase Na+/K+ Transporting Subunit Alpha 1
*ATP1B3*	ATPase Na+/K+ Transporting Subunit Beta 3
*AXL*	*AXL* Receptor Tyrosine Kinase
BAX	BCL2 Associated X, Apoptosis Regulator
*Bcl-2*	*B-cell lymphoma 2*
BRCP/ABCG2	breast cancer resistance protein
CADM1	cell adhesion molecule 1
CAFs	cancer-associated fibroblasts
*CALN1*	Calneuron 1
*CAV1*	Caveolin 1
CCAL	colorectal cancer-associated lncRNA
*CDKN1B*	Cyclin Dependent Kinase Inhibitor 1B
CEACAM	carcinoembryonic antigen related cell adhesion molecule
*CLIC1*	Chloride Intracellular Channel 1
CLIC1	protein chloride intracellular channel-1
CXCR-4	C-X-C chemokine receptor type 4
*DCK*	Deoxycytidine Kinase
DNMT1	DNA methyltranferase 1
DR	drug resistant
EGFR	*Epidermal growth factor receptor*
EMT	Epithelial to Mesenchymal Transition
*EPACAM*	Hepatic and Glial Cell Adhesion Molecule
EPHB2	ephrin type B receptor 2
*ERK*	extracellular-signal-regulated kinase
EVs	Extracellular Vesicles
FGF	fibroblast growth factor
GLUT-1	Glucose transporter 1
GSTP1	Glutathione S-transferase P
HER-2	Human Epidermal growth factor Receptor-type 2
*HMGA*	high mobility group A
*HNF1A*-*AS1*	HNF1A Antisense RNA 1
*HOTTIP*	HOXA Distal Transcript Antisense RNA
HSP70	Heat Shock Protein 70
IAP	Inhibitors of *apoptosis* proteins
ICAM-1	intercellular adhesion molecule 1
*ING5*	Inhibitor Of Growth Family Member 5
ISEV	International Society for Extracellular Vesicles
JNK	c-Jun N-terminal kinase
*LAMP*	lysosomal associated membrane *protein*
LncRNA	long non-coding RNAs
MAPK	*mitogen-activated protein kinase*
*MDM4*	*MDM4* Regulator of P53
MDR	multidrug resistance
*MECP2*	Methyl-CpG Binding Protein 2
miRs	microRNAs
MLH1	MutL homolog 1
MMPs	Matrix Metalloproteases
mRNAs	Messenger RNA
MRP1/ABCC1	multidrug resistance-associated protein 1
MSC	Mesenchymal Stromal Cells
MVs	ectosomes or microparticles,
NEU1	lysosomal sialidase
NFATc3	Nuclear Factor Of Activated T Cells 3
NSCLC	non-small cell lung cancer
PAX2	paired box gene 2
PDCD4	programmed cell death 4
PDGFR	platelet-derived growth factor
PDK	Protein 3-phosphoinositide-dependent protein kinase
PD-L1	Programmed death-ligand 1
P-gp/MDR1/ABCB1	P-glycoprotein
*PTPRZ1*	Protein Tyrosine Phosphatase Receptor Type Z1
*RAB7A*	*RAB7A*, Member RAS Oncogene Family
RBM11	RNA Binding Motif Protein 11
RhoA	member of the small GTPases family
*RIG*-*I*	retinoic acid-inducible gene I
ROR	Receptor tyrosine kinase-like orphan receptor
ROS	reactive oxygen species
*SBF2*-*AS1*	SBF2 antisense RNA 1
SNHG14	small nucleolar RNA host gene 14
SOD2	Superoxide dismutase 2
STAT3	*Signal transducer and activator of transcription 3*
*TERF1*	Telomeric Repeat Binding Factor 1
TERT	Telomerase reverse transcriptase
*TGFBR1*	Transforming Growth Factor Beta Receptor 1
TGF-β	transforming growth factor beta
*TGM2*	Transglutaminase 2
TIM-3	T-cell immunoglobulin and mucin-domain containing-3
TME	tumor microenvironment
*TP53INP1*	Tumor Protein P53 Inducible Nuclear Protein 1
TrpC5	Short transient receptor potential channel 5
TSG101	tumor susceptibility gene 101 protein
TSGA10	testis-specific gene antigen
*TUFT1*	Tuftelin 1
*VE*-*cadherin*	vascular endothelial cadherin
VEGF	*Vascular endothelial growth factor*
*VLDLR*	Very Low Density Lipoprotein Receptor
XIAP	X-linked inhibitor of apoptosis protein
XRCC4	X-ray repair cross-complementing protein 4
*ZEB1*-*AS1*	ZEB1 Antisense RNA 1

## References

[1] Thery C., Witwer K.W., Aikawa E., Alcaraz M.J., Anderson J.D., Andriantsitohaina R., Antoniou A., Arab T., Archer F., Atkin-Smith G.K. (2018). Minimal information for studies of extracellular vesicles 2018 (MISEV2018): A position statement of the International Society for Extracellular Vesicles and update of the MISEV2014 guidelines. J. Extracell. Vesicles.

[2] Roy S., Lin H.Y., Chou C.Y., Huang C.H., Small J., Sadik N., Ayinon C.M., Lansbury E., Cruz L., Yekula A. (2019). Navigating the Landscape of Tumor Extracellular Vesicle Heterogeneity. Int. J. Mol. Sci..

[3] Sousa D., Lima R.T., Vasconcelos M.H. (2015). Intercellular Transfer of Cancer Drug Resistance Traits by Extracellular Vesicles. Trends Mol. Med..

[4] Kim K.M., Abdelmohsen K., Mustapic M., Kapogiannis D., Gorospe M. (2017). RNA in extracellular vesicles. Wiley Interdiscip. Rev. RNA.

[5] Budnik V., Ruiz-Canada C., Wendler F. (2016). Extracellular vesicles round off communication in the nervous system. Nat. Rev. Neurosci..

[6] Yanez-Mo M., Siljander P.R., Andreu Z., Zavec A.B., Borras F.E., Buzas E.I., Buzas K., Casal E., Cappello F., Carvalho J. (2015). Biological properties of extracellular vesicles and their physiological functions. J. Extracell. Vesicles.

[7] Chargaff E., West R. (1946). The biological significance of the thromboplastic protein of blood. J. Biol. Chem..

[8] Wolf P. (1967). The nature and significance of platelet products in human plasma. Br. J. Haematol..

[9] Fruhbeis C., Frohlich D., Kuo W.P., Kramer-Albers E.M. (2013). Extracellular vesicles as mediators of neuron-glia communication. Front. Cell. Neurosci..

[10] Kosaka N., Iguchi H., Yoshioka Y., Hagiwara K., Takeshita F., Ochiya T. (2012). Competitive interactions of cancer cells and normal cells via secretory microRNAs. J. Biol. Chem..

[11] Puhka M., Takatalo M., Nordberg M.E., Valkonen S., Nandania J., Aatonen M., Yliperttula M., Laitinen S., Velagapudi V., Mirtti T. (2017). Metabolomic Profiling of Extracellular Vesicles and Alternative Normalization Methods Reveal Enriched Metabolites and Strategies to Study Prostate Cancer-Related Changes. Theranostics.

[12] Valadi H., Ekstrom K., Bossios A., Sjostrand M., Lee J.J., Lotvall J.O. (2007). Exosome-mediated transfer of mRNAs and microRNAs is a novel mechanism of genetic exchange between cells. Nat. Cell Biol..

[13] Vasconcelos M.H., Caires H.R., Abols A., Xavier C.P.R., Line A. (2019). Extracellular vesicles as a novel source of biomarkers in liquid biopsies for monitoring cancer progression and drug resistance. Drug Resist. Update.

[14] Thakur B.K., Zhang H., Becker A., Matei I., Huang Y., Costa-Silva B., Zheng Y., Hoshino A., Brazier H., Xiang J. (2014). Double-stranded DNA in exosomes: A novel biomarker in cancer detection. Cell Res..

[15] Kahlert C., Melo S.A., Protopopov A., Tang J., Seth S., Koch M., Zhang J., Weitz J., Chin L., Futreal A. (2014). Identification of double-stranded genomic DNA spanning all chromosomes with mutated KRAS and p53 DNA in the serum exosomes of patients with pancreatic cancer. J. Biol. Chem..

[16] Deregibus M.C., Cantaluppi V., Calogero R., Lo Iacono M., Tetta C., Biancone L., Bruno S., Bussolati B., Camussi G. (2007). Endothelial progenitor cell derived microvesicles activate an angiogenic program in endothelial cells by a horizontal transfer of mRNA. Blood.

[17] Fang T., Lv H., Lv G., Li T., Wang C., Han Q., Yu L., Su B., Guo L., Huang S. (2018). Tumor-derived exosomal miR-1247-3p induces cancer-associated fibroblast activation to foster lung metastasis of liver cancer. Nat. Commun..

[18] Hoshino A., Costa-Silva B., Shen T.L., Rodrigues G., Hashimoto A., Tesic Mark M., Molina H., Kohsaka S., Di Giannatale A., Ceder S. (2015). Tumour exosome integrins determine organotropic metastasis. Nature.

[19] Peinado H., Aleckovic M., Lavotshkin S., Matei I., Costa-Silva B., Moreno-Bueno G., Hergueta-Redondo M., Williams C., Garcia-Santos G., Ghajar C. (2012). Melanoma exosomes educate bone marrow progenitor cells toward a pro-metastatic phenotype through MET. Nat. Med..

[20] Peinado H., Zhang H., Matei I.R., Costa-Silva B., Hoshino A., Rodrigues G., Psaila B., Kaplan R.N., Bromberg J.F., Kang Y. (2017). Pre-metastatic niches: Organ-specific homes for metastases. Nat. Rev. Cancer.

[21] Caby M.P., Lankar D., Vincendeau-Scherrer C., Raposo G., Bonnerot C. (2005). Exosomal-like vesicles are present in human blood plasma. Int. Immunol..

[22] Arraud N., Linares R., Tan S., Gounou C., Pasquet J.M., Mornet S., Brisson A.R. (2014). Extracellular vesicles from blood plasma: Determination of their morphology, size, phenotype and concentration. J. Thromb. Haemost..

[23] Aalberts M., van Dissel-Emiliani F.M., van Adrichem N.P., van Wijnen M., Wauben M.H., Stout T.A., Stoorvogel W. (2012). Identification of distinct populations of prostasomes that differentially express prostate stem cell antigen, annexin A1, and GLIPR2 in humans. Biol. Reprod..

[24] Gonzales P.A., Zhou H., Pisitkun T., Wang N.S., Star R.A., Knepper M.A., Yuen P.S. (2010). Isolation and purification of exosomes in urine. Methods Mol. Biol..

[25] Admyre C., Johansson S.M., Qazi K.R., Filen J.J., Lahesmaa R., Norman M., Neve E.P., Scheynius A., Gabrielsson S. (2007). Exosomes with immune modulatory features are present in human breast milk. J. Immunol..

[26] Lasser C., O’Neil S.E., Ekerljung L., Ekstrom K., Sjostrand M., Lotvall J. (2011). RNA-containing exosomes in human nasal secretions. Am. J. Rhinol. Allergy.

[27] Palanisamy V., Sharma S., Deshpande A., Zhou H., Gimzewski J., Wong D.T. (2010). Nanostructural and transcriptomic analyses of human saliva derived exosomes. PLoS ONE.

[28] Koga Y., Yasunaga M., Moriya Y., Akasu T., Fujita S., Yamamoto S., Matsumura Y. (2011). Exosome can prevent RNase from degrading microRNA in feces. J. Gastrointest. Oncol..

[29] Armstrong D., Wildman D.E. (2018). Extracellular Vesicles and the Promise of Continuous Liquid Biopsies. J. Pathol. Transl. Med..

[30] Yokoi A., Yoshioka Y., Yamamoto Y., Ishikawa M., Ikeda S.I., Kato T., Kiyono T., Takeshita F., Kajiyama H., Kikkawa F. (2017). Malignant extracellular vesicles carrying MMP1 mRNA facilitate peritoneal dissemination in ovarian cancer. Nat. Commun..

[31] Muralidharan-Chari V., Clancy J., Plou C., Romao M., Chavrier P., Raposo G., D’Souza-Schorey C. (2009). ARF6-regulated shedding of tumor cell-derived plasma membrane microvesicles. Curr. Biol..

[32] Caruso Bavisotto C., Scalia F., Marino Gammazza A., Carlisi D., Bucchieri F., Conway de Macario E., Macario A.J.L., Cappello F., Campanella C. (2019). Extracellular Vesicle-Mediated Cell(-)Cell Communication in the Nervous System: Focus on Neurological Diseases. Int. J. Mol. Sci..

[33] Doyle L.M., Wang M.Z. (2019). Overview of Extracellular Vesicles, Their Origin, Composition, Purpose, and Methods for Exosome Isolation and Analysis. Cells.

[34] Perut F., Roncuzzi L., Baldini N. (2019). The Emerging Roles of Extracellular Vesicles in Osteosarcoma. Front. Oncol..

[35] Graves L.E., Ariztia E.V., Navari J.R., Matzel H.J., Stack M.S., Fishman D.A. (2004). Proinvasive properties of ovarian cancer ascites-derived membrane vesicles. Cancer Res..

[36] Menck K., Bleckmann A., Wachter A., Hennies B., Ries L., Schulz M., Balkenhol M., Pukrop T., Schatlo B., Rost U. (2017). Characterisation of tumour-derived microvesicles in cancer patients’ blood and correlation with clinical outcome. J. Extracell. Vesicles.

[37] Hallal S., Russell B.P., Wei H., Lee M.Y.T., Toon C.W., Sy J., Shivalingam B., Buckland M.E., Kaufman K.L. (2019). Extracellular Vesicles from Neurosurgical Aspirates Identifies Chaperonin Containing TCP1 Subunit 6A as a Potential Glioblastoma Biomarker with Prognostic Significance. Proteomics.

[38] Sedlarikova L., Bollova B., Radova L., Brozova L., Jarkovsky J., Almasi M., Penka M., Kuglik P., Sandecka V., Stork M. (2018). Circulating exosomal long noncoding RNA PRINS-First findings in monoclonal gammopathies. Hematol. Oncol..

[39] Yadav D.K., Bai X., Yadav R.K., Singh A., Li G., Ma T., Chen W., Liang T. (2018). Liquid biopsy in pancreatic cancer: The beginning of a new era. Oncotarget.

[40] Yuana Y., Sturk A., Nieuwland R. (2013). Extracellular vesicles in physiological and pathological conditions. Blood Rev..

[41] Claridge B., Kastaniegaard K., Stensballe A., Greening D.W. (2019). Post-translational and transcriptional dynamics—Regulating extracellular vesicle biology. Expert Rev. Proteom..

[42] Smith J.A., Leonardi T., Huang B., Iraci N., Vega B., Pluchino S. (2015). Extracellular vesicles and their synthetic analogues in aging and age-associated brain diseases. Biogerontology.

[43] Baek R., Sondergaard E.K., Varming K., Jorgensen M.M. (2016). The impact of various preanalytical treatments on the phenotype of small extracellular vesicles in blood analyzed by protein microarray. J. Immunol. Methods.

[44] Bjorge I.M., Kim S.Y., Mano J.F., Kalionis B., Chrzanowski W. (2017). Extracellular vesicles, exosomes and shedding vesicles in regenerative medicine—A new paradigm for tissue repair. Biomater. Sci..

[45] Syn N.L., Wang L., Chow E.K., Lim C.T., Goh B.C. (2017). Exosomes in Cancer Nanomedicine and Immunotherapy: Prospects and Challenges. Trends Biotechnol..

[46] Zhupanyn P., Ewe A., Buch T., Malek A., Rademacher P., Muller C., Reinert A., Jaimes Y., Aigner A. (2020). Extracellular vesicle (ECV)-modified polyethylenimine (PEI) complexes for enhanced siRNA delivery in vitro and in vivo. J. Control. Release.

[47] Elsharkasy O.M., Nordin J.Z., Hagey D.W., de Jong O.G., Schiffelers R.M., Andaloussi S.E.L., Vader P. (2020). Extracellular vesicles as drug delivery systems: Why and how?. Adv. Drug Deliv. Rev..

[48] Pavlyukov M.S., Yu H., Bastola S., Minata M., Shender V.O., Lee Y., Zhang S., Wang J., Komarova S., Wang J. (2018). Apoptotic Cell-Derived Extracellular Vesicles Promote Malignancy of Glioblastoma Via Intercellular Transfer of Splicing Factors. Cancer Cell.

[49] Setti M., Osti D., Richichi C., Ortensi B., Del Bene M., Fornasari L., Beznoussenko G., Mironov A., Rappa G., Cuomo A. (2015). Extracellular vesicle-mediated transfer of CLIC1 protein is a novel mechanism for the regulation of glioblastoma growth. Oncotarget.

[50] Vella L.J., Behren A., Coleman B., Greening D.W., Hill A.F., Cebon J. (2017). Intercellular Resistance to BRAF Inhibition Can Be Mediated by Extracellular Vesicle-Associated PDGFRbeta. Neoplasia.

[51] Jin H., Liu P., Wu Y., Meng X., Wu M., Han J., Tan X. (2018). Exosomal zinc transporter ZIP4 promotes cancer growth and is a novel diagnostic biomarker for pancreatic cancer. Cancer Sci..

[52] Liu M.X., Liao J., Xie M., Gao Z.K., Wang X.H., Zhang Y., Shang M.H., Yin L.H., Pu Y.P., Liu R. (2018). miR-93-5p Transferred by Exosomes Promotes the Proliferation of Esophageal Cancer Cells via Intercellular Communication by Targeting PTEN. Biomed. Environ. Sci..

[53] Chen D., Wu X., Xia M., Wu F., Ding J., Jiao Y., Zhan Q., An F. (2017). Upregulated exosomic miR23b3p plays regulatory roles in the progression of pancreatic cancer. Oncol. Rep..

[54] Li X.J., Ren Z.J., Tang J.H., Yu Q. (2017). Exosomal MicroRNA MiR-1246 Promotes Cell Proliferation, Invasion and Drug Resistance by Targeting CCNG2 in Breast Cancer. Cell Physiol. Biochem..

[55] Kitdumrongthum S., Metheetrairut C., Charoensawan V., Ounjai P., Janpipatkul K., Panvongsa W., Weerachayaphorn J., Piyachaturawat P., Chairoungdua A. (2018). Dysregulated microRNA expression profiles in cholangiocarcinoma cell-derived exosomes. Life Sci..

[56] Soldevilla B., Rodriguez M., San Millan C., Garcia V., Fernandez-Perianez R., Gil-Calderon B., Martin P., Garcia-Grande A., Silva J., Bonilla F. (2014). Tumor-derived exosomes are enriched in DeltaNp73, which promotes oncogenic potential in acceptor cells and correlates with patient survival. Hum. Mol. Genet..

[57] Teng Y., Ren Y., Hu X., Mu J., Samykutty A., Zhuang X., Deng Z., Kumar A., Zhang L., Merchant M.L. (2017). MVP-mediated exosomal sorting of miR-193a promotes colon cancer progression. Nat. Commun..

[58] Zhang Z., Xing T., Chen Y., Xiao J. (2018). Exosome-mediated miR-200b promotes colorectal cancer proliferation upon TGF-beta1 exposure. Biomed. Pharmacother..

[59] Guo K., Yao J., Yu Q., Li Z., Huang H., Cheng J., Wang Z., Zhu Y. (2017). The expression pattern of long non-coding RNA PVT1 in tumor tissues and in extracellular vesicles of colorectal cancer correlates with cancer progression. Tumour. Biol..

[60] Lee J.C., Zhao J.T., Gundara J., Serpell J., Bach L.A., Sidhu S. (2015). Papillary thyroid cancer-derived exosomes contain miRNA-146b and miRNA-222. J. Surg. Res..

[61] Cappellesso R., Tinazzi A., Giurici T., Simonato F., Guzzardo V., Ventura L., Crescenzi M., Chiarelli S., Fassina A. (2014). Programmed cell death 4 and microRNA 21 inverse expression is maintained in cells and exosomes from ovarian serous carcinoma effusions. Cancer Cytopathol..

[62] Lu L., Chen X.M., Tao H.M., Xiong W., Jie S.H., Li H.Y. (2015). Regulation of the expression of zinc finger protein genes by microRNAs enriched within acute lymphoblastic leukemia-derived microvesicles. Genet. Mol. Res..

[63] Pan L., Liang W., Fu M., Huang Z.H., Li X., Zhang W., Zhang P., Qian H., Jiang P.C., Xu W.R. (2017). Exosomes-mediated transfer of long noncoding RNA ZFAS1 promotes gastric cancer progression. J. Cancer Res. Clin. Oncol..

[64] Zhang Y.G., Zhou M.W., Bai L., Han R.Y., Lv K., Wang Z. (2018). Extracellular vesicles promote esophageal cancer progression by delivering lncZEB1-AS1 between cells. Eur. Rev. Med. Pharmacol. Sci..

[65] Kogure T., Yan I.K., Lin W.L., Patel T. (2013). Extracellular Vesicle-Mediated Transfer of a Novel Long Noncoding RNA TUC339: A Mechanism of Intercellular Signaling in Human Hepatocellular Cancer. Genes Cancer.

[66] Lang H.L., Hu G.W., Zhang B., Kuang W., Chen Y., Wu L., Xu G.H. (2017). Glioma cells enhance angiogenesis and inhibit endothelial cell apoptosis through the release of exosomes that contain long non-coding RNA CCAT2. Oncol. Rep..

[67] Lang H.L., Hu G.W., Chen Y., Liu Y., Tu W., Lu Y.M., Wu L., Xu G.H. (2017). Glioma cells promote angiogenesis through the release of exosomes containing long non-coding RNA POU3F3. Eur. Rev. Med. Pharmacol. Sci..

[68] Sun X., Ma X., Wang J., Zhao Y., Wang Y., Bihl J.C., Chen Y., Jiang C. (2017). Glioma stem cells-derived exosomes promote the angiogenic ability of endothelial cells through miR-21/VEGF signal. Oncotarget.

[69] Giusti I., Delle Monache S., Di Francesco M., Sanita P., D’Ascenzo S., Gravina G.L., Festuccia C., Dolo V. (2016). From glioblastoma to endothelial cells through extracellular vesicles: Messages for angiogenesis. Tumour. Biol..

[70] Sato S., Vasaikar S., Eskaros A., Kim Y., Lewis J.S., Zhang B., Zijlstra A., Weaver A.M. (2019). EPHB2 carried on small extracellular vesicles induces tumor angiogenesis via activation of ephrin reverse signaling. JCI Insight.

[71] Huang A., Dong J., Li S., Wang C., Ding H., Li H., Su X., Ge X., Sun L., Bai C. (2015). Exosomal transfer of vasorin expressed in hepatocellular carcinoma cells promotes migration of human umbilical vein endothelial cells. Int. J. Biol. Sci..

[72] Fang J.H., Zhang Z.J., Shang L.R., Luo Y.W., Lin Y.F., Yuan Y., Zhuang S.M. (2018). Hepatoma cell-secreted exosomal microRNA-103 increases vascular permeability and promotes metastasis by targeting junction proteins. Hepatology.

[73] Li B., Hong J., Hong M., Wang Y., Yu T., Zang S., Wu Q. (2019). piRNA-823 delivered by multiple myeloma-derived extracellular vesicles promoted tumorigenesis through re-educating endothelial cells in the tumor environment. Oncogene.

[74] Zeng Z., Li Y., Pan Y., Lan X., Song F., Sun J., Zhou K., Liu X., Ren X., Wang F. (2018). Cancer-derived exosomal miR-25-3p promotes pre-metastatic niche formation by inducing vascular permeability and angiogenesis. Nat. Commun..

[75] Lawson J., Dickman C., MacLellan S., Towle R., Jabalee J., Lam S., Garnis C. (2017). Selective secretion of microRNAs from lung cancer cells via extracellular vesicles promotes CAMK1D-mediated tube formation in endothelial cells. Oncotarget.

[76] Zheng Y., Liu L., Chen C., Ming P., Huang Q., Li C., Cao D., Xu X., Ge W. (2017). The extracellular vesicles secreted by lung cancer cells in radiation therapy promote endothelial cell angiogenesis by transferring miR-23a. PeerJ.

[77] Masoumi-Dehghi S., Babashah S., Sadeghizadeh M. (2020). MicroRNA-141-3p-containing small extracellular vesicles derived from epithelial ovarian cancer cells promote endothelial cell angiogenesis through activating the JAK/STAT3 and NF-kappaB signaling pathways. J. Cell Commun. Signal..

[78] Dickman C.T., Lawson J., Jabalee J., MacLellan S.A., LePard N.E., Bennewith K.L., Garnis C. (2017). Selective extracellular vesicle exclusion of miR-142-3p by oral cancer cells promotes both internal and extracellular malignant phenotypes. Oncotarget.

[79] Bao L., You B., Shi S., Shan Y., Zhang Q., Yue H., Zhang J., Zhang W., Shi Y., Liu Y. (2018). Metastasis-associated miR-23a from nasopharyngeal carcinoma-derived exosomes mediates angiogenesis by repressing a novel target gene TSGA10. Oncogene.

[80] Campos A., Salomon C., Bustos R., Diaz J., Martinez S., Silva V., Reyes C., Diaz-Valdivia N., Varas-Godoy M., Lobos-Gonzalez L. (2018). Caveolin-1-containing extracellular vesicles transport adhesion proteins and promote malignancy in breast cancer cell lines. Nanomedicine (Lond.).

[81] Harada T., Yamamoto H., Kishida S., Kishida M., Awada C., Takao T., Kikuchi A. (2017). Wnt5b-associated exosomes promote cancer cell migration and proliferation. Cancer Sci..

[82] Higginbotham J.N., Demory Beckler M., Gephart J.D., Franklin J.L., Bogatcheva G., Kremers G.J., Piston D.W., Ayers G.D., McConnell R.E., Tyska M.J. (2011). Amphiregulin exosomes increase cancer cell invasion. Curr. Biol..

[83] Qu Z., Feng J., Pan H., Jiang Y., Duan Y., Fa Z. (2019). Exosomes derived from HCC cells with different invasion characteristics mediated EMT through TGF-beta/Smad signaling pathway. Oncol. Targets Ther..

[84] Li M., Lu Y., Xu Y., Wang J., Zhang C., Du Y., Wang L., Li L., Wang B., Shen J. (2018). Horizontal transfer of exosomal CXCR4 promotes murine hepatocarcinoma cell migration, invasion and lymphangiogenesis. Gene.

[85] Fu Q., Zhang Q., Lou Y., Yang J., Nie G., Chen Q., Chen Y., Zhang J., Wang J., Wei T. (2018). Primary tumor-derived exosomes facilitate metastasis by regulating adhesion of circulating tumor cells via SMAD3 in liver cancer. Oncogene.

[86] Xue X., Wang X., Zhao Y., Hu R., Qin L. (2018). Exosomal miR-93 promotes proliferation and invasion in hepatocellular carcinoma by directly inhibiting TIMP2/TP53INP1/CDKN1A. Biochem. Biophys. Res. Commun..

[87] Yang H., Fu H., Wang B., Zhang X., Mao J., Li X., Wang M., Sun Z., Qian H., Xu W. (2018). Exosomal miR-423-5p targets SUFU to promote cancer growth and metastasis and serves as a novel marker for gastric cancer. Mol. Carcinog..

[88] Bhagirath D., Yang T.L., Bucay N., Sekhon K., Majid S., Shahryari V., Dahiya R., Tanaka Y., Saini S. (2018). microRNA-1246 Is an Exosomal Biomarker for Aggressive Prostate Cancer. Cancer Res..

[89] Cai Q., Zhu A., Gong L. (2018). Exosomes of glioma cells deliver miR-148a to promote proliferation and metastasis of glioblastoma via targeting CADM1. Bull. Cancer.

[90] Yoshimura A., Sawada K., Nakamura K., Kinose Y., Nakatsuka E., Kobayashi M., Miyamoto M., Ishida K., Matsumoto Y., Kodama M. (2018). Exosomal miR-99a-5p is elevated in sera of ovarian cancer patients and promotes cancer cell invasion by increasing fibronectin and vitronectin expression in neighboring peritoneal mesothelial cells. BMC Cancer.

[91] Yang S.J., Wang D.D., Li J., Xu H.Z., Shen H.Y., Chen X., Zhou S.Y., Zhong S.L., Zhao J.H., Tang J.H. (2017). Predictive role of GSTP1-containing exosomes in chemotherapy-resistant breast cancer. Gene.

[92] Fong M.Y., Zhou W., Liu L., Alontaga A.Y., Chandra M., Ashby J., Chow A., O’Connor S.T., Li S., Chin A.R. (2015). Breast-cancer-secreted miR-122 reprograms glucose metabolism in premetastatic niche to promote metastasis. Nat. Cell Biol..

[93] Zeng A.L., Yan W., Liu Y.W., Wang Z., Hu Q., Nie E., Zhou X., Li R., Wang X.F., Jiang T. (2017). Tumour exosomes from cells harbouring PTPRZ1-MET fusion contribute to a malignant phenotype and temozolomide chemoresistance in glioblastoma. Oncogene.

[94] Al-Nedawi K., Meehan B., Micallef J., Lhotak V., May L., Guha A., Rak J. (2008). Intercellular transfer of the oncogenic receptor EGFRvIII by microvesicles derived from tumour cells. Nat. Cell Biol..

[95] Cesi G., Philippidou D., Kozar I., Kim Y.J., Bernardin F., Van Niel G., Wienecke-Baldacchino A., Felten P., Letellier E., Dengler S. (2018). A new ALK isoform transported by extracellular vesicles confers drug resistance to melanoma cells. Mol. Cancer.

[96] Kalra H., Gangoda L., Fonseka P., Chitti S.V., Liem M., Keerthikumar S., Samuel M., Boukouris S., Al Saffar H., Collins C. (2019). Extracellular vesicles containing oncogenic mutant beta-catenin activate Wnt signalling pathway in the recipient cells. J. Extracell. Vesicles.

[97] Crow J., Atay S., Banskota S., Artale B., Schmitt S., Godwin A.K. (2017). Exosomes as mediators of platinum resistance in ovarian cancer. Oncotarget.

[98] Kanlikilicer P., Bayraktar R., Denizli M., Rashed M.H., Ivan C., Aslan B., Mitra R., Karagoz K., Bayraktar E., Zhang X. (2018). Exosomal miRNA confers chemo resistance via targeting Cav1/p-gp/M2-type macrophage axis in ovarian cancer. EBioMedicine.

[99] Czystowska-Kuzmicz M., Sosnowska A., Nowis D., Ramji K., Szajnik M., Chlebowska-Tuz J., Wolinska E., Gaj P., Grazul M., Pilch Z. (2019). Small extracellular vesicles containing arginase-1 suppress T-cell responses and promote tumor growth in ovarian carcinoma. Nat. Commun..

[100] Cooks T., Pateras I.S., Jenkins L.M., Patel K.M., Robles A.I., Morris J., Forshew T., Appella E., Gorgoulis V.G., Harris C.C. (2018). Mutant p53 cancers reprogram macrophages to tumor supporting macrophages via exosomal miR-1246. Nat. Commun..

[101] Muturi H.T., Dreesen J.D., Nilewski E., Jastrow H., Giebel B., Ergun S., Singer B.B. (2013). Tumor and endothelial cell-derived microvesicles carry distinct CEACAMs and influence T-cell behavior. PLoS ONE.

[102] Dai G., Yao X., Zhang Y., Gu J., Geng Y., Xue F., Zhang J. (2018). Colorectal cancer cell-derived exosomes containing miR-10b regulate fibroblast cells via the PI3K/Akt pathway. Bull. Cancer.

[103] Casadei L., Calore F., Creighton C.J., Guescini M., Batte K., Iwenofu O.H., Zewdu A., Braggio D.A., Bill K.L., Fadda P. (2017). Exosome-Derived miR-25-3p and miR-92a-3p Stimulate Liposarcoma Progression. Cancer Res..

[104] van der Vos K.E., Abels E.R., Zhang X., Lai C., Carrizosa E., Oakley D., Prabhakar S., Mardini O., Crommentuijn M.H., Skog J. (2015). Directly visualized glioblastoma-derived extracellular vesicles transfer RNA to microglia/macrophages in the brain. Neuro. Oncol..

[105] Ricklefs F.L., Alayo Q., Krenzlin H., Mahmoud A.B., Speranza M.C., Nakashima H., Hayes J.L., Lee K., Balaj L., Passaro C. (2018). Immune evasion mediated by PD-L1 on glioblastoma-derived extracellular vesicles. Sci. Adv..

[106] Li X., Lei Y., Wu M., Li N. (2018). Regulation of Macrophage Activation and Polarization by HCC-Derived Exosomal lncRNA TUC339. Int. J. Mol. Sci..

[107] Zhou Y., Ren H., Dai B., Li J., Shang L., Huang J., Shi X. (2018). Hepatocellular carcinoma-derived exosomal miRNA-21 contributes to tumor progression by converting hepatocyte stellate cells to cancer-associated fibroblasts. J. Exp. Clin. Cancer Res..

[108] Ye S.B., Zhang H., Cai T.T., Liu Y.N., Ni J.J., He J., Peng J.Y., Chen Q.Y., Mo H.Y., Jun C. (2016). Exosomal miR-24-3p impedes T-cell function by targeting FGF11 and serves as a potential prognostic biomarker for nasopharyngeal carcinoma. J. Pathol..

[109] Ye S.B., Li Z.L., Luo D.H., Huang B.J., Chen Y.S., Zhang X.S., Cui J., Zeng Y.X., Li J. (2014). Tumor-derived exosomes promote tumor progression and T-cell dysfunction through the regulation of enriched exosomal microRNAs in human nasopharyngeal carcinoma. Oncotarget.

[110] Klibi J., Niki T., Riedel A., Pioche-Durieu C., Souquere S., Rubinstein E., Le Moulec S., Guigay J., Hirashima M., Guemira F. (2009). Blood diffusion and Th1-suppressive effects of galectin-9-containing exosomes released by Epstein-Barr virus-infected nasopharyngeal carcinoma cells. Blood.

[111] Yang L., Wu X., Wang D., Luo C., Chen L. (2013). Renal carcinoma cell-derived exosomes induce human immortalized line of Jurkat T lymphocyte apoptosis in vitro. Urol. Int..

[112] Gong L., Bao Q., Hu C., Wang J., Zhou Q., Wei L., Tong L., Zhang W., Shen Y. (2018). Exosomal miR-675 from metastatic osteosarcoma promotes cell migration and invasion by targeting CALN1. Biochem. Biophys. Res. Commun..

[113] Wang J., Guan X., Zhang Y., Ge S., Zhang L., Li H., Wang X., Liu R., Ning T., Deng T. (2018). Exosomal miR-27a Derived from Gastric Cancer Cells Regulates the Transformation of Fibroblasts into Cancer-Associated Fibroblasts. Cell Physiol. Biochem..

[114] Zhou X., Yan T., Huang C., Xu Z., Wang L., Jiang E., Wang H., Chen Y., Liu K., Shao Z. (2018). Melanoma cell-secreted exosomal miR-155-5p induce proangiogenic switch of cancer-associated fibroblasts via SOCS1/JAK2/STAT3 signaling pathway. J. Exp. Clin. Cancer Res..

[115] Lawson J., Dickman C., Towle R., Jabalee J., Javer A., Garnis C. (2019). Extracellular vesicle secretion of miR-142-3p from lung adenocarcinoma cells induces tumor promoting changes in the stroma through cell-cell communication. Mol. Carcinog..

[116] Yang L., Wu X.H., Wang D., Luo C.L., Chen L.X. (2013). Bladder cancer cell-derived exosomes inhibit tumor cell apoptosis and induce cell proliferation in vitro. Mol. Med. Rep..

[117] Qu J.L., Qu X.J., Zhao M.F., Teng Y.E., Zhang Y., Hou K.Z., Jiang Y.H., Yang X.H., Liu Y.P. (2009). Gastric cancer exosomes promote tumour cell proliferation through PI3K/Akt and MAPK/ERK activation. Dig. Liver Dis..

[118] Li Z., Tao Y., Wang X., Jiang P., Li J., Peng M., Zhang X., Chen K., Liu H., Zhen P. (2018). Tumor-Secreted Exosomal miR-222 Promotes Tumor Progression via Regulating P27 Expression and Re-Localization in Pancreatic Cancer. Cell Physiol. Biochem..

[119] Li C., Liu D.R., Li G.G., Wang H.H., Li X.W., Zhang W., Wu Y.L., Chen L. (2015). CD97 promotes gastric cancer cell proliferation and invasion through exosome-mediated MAPK signaling pathway. World J. Gastroenterol..

[120] Ghorbanian M., Babashah S., Ataei F. (2019). The effects of ovarian cancer cell-derived exosomes on vascular endothelial growth factor expression in endothelial cells. EXCLI J..

[121] Lu J., Liu Q.H., Wang F., Tan J.J., Deng Y.Q., Peng X.H., Liu X., Zhang B., Xu X., Li X.P. (2018). Exosomal miR-9 inhibits angiogenesis by targeting MDK and regulating PDK/AKT pathway in nasopharyngeal carcinoma. J. Exp. Clin. Cancer Res..

[122] de Andrade A., de Oliveira C.E., Dourado M.R., Macedo C., Winck F.V., Paes Leme A.F., Salo T., Coletta R.D., de Almeida Freitas R., Galvao H.C. (2018). Extracellular vesicles from oral squamous carcinoma cells display pro- and anti-angiogenic properties. Oral. Dis..

[123] Souza A.G., IB B.S., Campos-Fernandez E., Marangoni K., VA F.B., Alves P.T., Goulart L.R., Alonso-Goulart V. (2018). Extracellular vesicles as drivers of epithelial-mesenchymal transition and carcinogenic characteristics in normal prostate cells. Mol. Carcinog..

[124] El-Sayed I.Y., Daher A., Destouches D., Firlej V., Kostallari E., Maille P., Huet E., Haidar-Ahmad N., Jenster G., de la Taille A. (2017). Extracellular vesicles released by mesenchymal-like prostate carcinoma cells modulate EMT state of recipient epithelial-like carcinoma cells through regulation of AR signaling. Cancer Lett..

[125] Brzozowski J.S., Bond D.R., Jankowski H., Goldie B.J., Burchell R., Naudin C., Smith N.D., Scarlett C.J., Larsen M.R., Dun M.D. (2018). Extracellular vesicles with altered tetraspanin CD9 and CD151 levels confer increased prostate cell motility and invasion. Sci. Rep..

[126] Jiang X.L., Zhang Y., Tan B., Luo C.L., Wu X.H. (2014). Renal tumor-derived exosomes inhibit hepaCAM expression of renal carcinoma cells in a p-AKT-dependent manner. Neoplasma.

[127] Chiba M., Watanabe N., Watanabe M., Sakamoto M., Sato A., Fujisaki M., Kubota S., Monzen S., Maruyama A., Nanashima N. (2016). Exosomes derived from SW480 colorectal cancer cells promote cell migration in HepG2 hepatocellular cancer cells via the mitogen-activated protein kinase pathway. Int. J. Oncol..

[128] Lopes-Rodrigues V., Di Luca A., Mleczko J., Meleady P., Henry M., Pesic M., Cabrera D., van Liempd S., Lima R.T., O’Connor R. (2017). Identification of the metabolic alterations associated with the multidrug resistant phenotype in cancer and their intercellular transfer mediated by extracellular vesicles. Sci. Rep..

[129] Kawamura Y., Yamamoto Y., Sato T.A., Ochiya T. (2017). Extracellular vesicles as trans-genomic agents: Emerging roles in disease and evolution. Cancer Sci..

[130] Guo D., Chen Y., Wang S., Yu L., Shen Y., Zhong H., Yang Y. (2018). Exosomes from heat-stressed tumour cells inhibit tumour growth by converting regulatory T cells to Th17 cells via IL-6. Immunology.

[131] Bu N., Li Q.L., Feng Q., Sun B.Z. (2006). Immune protection effect of exosomes against attack of L1210 tumor cells. Leuk. Lymphoma.

[132] Chen X., Ying X., Wang X., Wu X., Zhu Q., Wang X. (2017). Exosomes derived from hypoxic epithelial ovarian cancer deliver microRNA-940 to induce macrophage M2 polarization. Oncol. Rep..

[133] Marton A., Vizler C., Kusz E., Temesfoi V., Szathmary Z., Nagy K., Szegletes Z., Varo G., Siklos L., Katona R.L. (2012). Melanoma cell-derived exosomes alter macrophage and dendritic cell functions in vitro. Immunol. Lett..

[134] Yamada N., Kuranaga Y., Kumazaki M., Shinohara H., Taniguchi K., Akao Y. (2016). Colorectal cancer cell-derived extracellular vesicles induce phenotypic alteration of T cells into tumor-growth supporting cells with transforming growth factor-beta1-mediated suppression. Oncotarget.

[135] Rong L., Li R., Li S., Luo R. (2016). Immunosuppression of breast cancer cells mediated by transforming growth factor-beta in exosomes from cancer cells. Oncol. Lett..

[136] Huber V., Fais S., Iero M., Lugini L., Canese P., Squarcina P., Zaccheddu A., Colone M., Arancia G., Gentile M. (2005). Human colorectal cancer cells induce T-cell death through release of proapoptotic microvesicles: Role in immune escape. Gastroenterology.

[137] Andreola G., Rivoltini L., Castelli C., Huber V., Perego P., Deho P., Squarcina P., Accornero P., Lozupone F., Lugini L. (2002). Induction of lymphocyte apoptosis by tumor cell secretion of FasL-bearing microvesicles. J. Exp. Med..

[138] Abusamra A.J., Zhong Z., Zheng X., Li M., Ichim T.E., Chin J.L., Min W.P. (2005). Tumor exosomes expressing Fas ligand mediate CD8+ T-cell apoptosis. Blood Cells Mol. Dis..

[139] Kim J.W., Wieckowski E., Taylor D.D., Reichert T.E., Watkins S., Whiteside T.L. (2005). Fas ligand-positive membranous vesicles isolated from sera of patients with oral cancer induce apoptosis of activated T lymphocytes. Clin. Cancer Res..

[140] Li Y., An J., Huang S., He J., Zhang J. (2015). Esophageal cancer-derived microvesicles induce regulatory B cells. Cell Biochem. Funct..

[141] Paggetti J., Haderk F., Seiffert M., Janji B., Distler U., Ammerlaan W., Kim Y.J., Adam J., Lichter P., Solary E. (2015). Exosomes released by chronic lymphocytic leukemia cells induce the transition of stromal cells into cancer-associated fibroblasts. Blood.

[142] Giusti I., Di Francesco M., D’Ascenzo S., Palmerini M.G., Macchiarelli G., Carta G., Dolo V. (2018). Ovarian cancer-derived extracellular vesicles affect normal human fibroblast behavior. Cancer Biol. Ther..

[143] Dorsam B., Bosl T., Reiners K.S., Barnert S., Schubert R., Shatnyeva O., Zigrino P., Engert A., Hansen H.P., von Strandmann E.P. (2018). Hodgkin Lymphoma-Derived Extracellular Vesicles Change the Secretome of Fibroblasts Toward a CAF Phenotype. Front. Immunol..

[144] Ning X., Zhang H., Wang C., Song X. (2018). Exosomes Released by Gastric Cancer Cells Induce Transition of Pericytes Into Cancer-Associated Fibroblasts. Med. Sci. Monit..

[145] Pang W., Su J., Wang Y., Feng H., Dai X., Yuan Y., Chen X., Yao W. (2015). Pancreatic cancer-secreted miR-155 implicates in the conversion from normal fibroblasts to cancer-associated fibroblasts. Cancer Sci..

[146] Bouvy C., Wannez A., Laloy J., Chatelain C., Dogne J.M. (2017). Transfer of multidrug resistance among acute myeloid leukemia cells via extracellular vesicles and their microRNA cargo. Leuk. Res..

[147] Aung T., Chapuy B., Vogel D., Wenzel D., Oppermann M., Lahmann M., Weinhage T., Menck K., Hupfeld T., Koch R. (2011). Exosomal evasion of humoral immunotherapy in aggressive B-cell lymphoma modulated by ATP-binding cassette transporter A3. Proc. Natl. Acad. Sci. USA.

[148] Corcoran C., Rani S., O’Brien K., O’Neill A., Prencipe M., Sheikh R., Webb G., McDermott R., Watson W., Crown J. (2012). Docetaxel-resistance in prostate cancer: Evaluating associated phenotypic changes and potential for resistance transfer via exosomes. PLoS ONE.

[149] Kharaziha P., Chioureas D., Rutishauser D., Baltatzis G., Lennartsson L., Fonseca P., Azimi A., Hultenby K., Zubarev R., Ullen A. (2015). Molecular profiling of prostate cancer derived exosomes may reveal a predictive signature for response to docetaxel. Oncotarget.

[150] Lv M.M., Zhu X.Y., Chen W.X., Zhong S.L., Hu Q., Ma T.F., Zhang J., Chen L., Tang J.H., Zhao J.H. (2014). Exosomes mediate drug resistance transfer in MCF-7 breast cancer cells and a probable mechanism is delivery of P-glycoprotein. Tumour. Biol..

[151] Ning K., Wang T., Sun X., Zhang P., Chen Y., Jin J., Hua D. (2017). UCH-L1-containing exosomes mediate chemotherapeutic resistance transfer in breast cancer. J. Surg. Oncol..

[152] Dong Y., Pan Q., Jiang L., Chen Z., Zhang F., Liu Y., Xing H., Shi M., Li J., Li X. (2014). Tumor endothelial expression of P-glycoprotein upon microvesicular transfer of TrpC5 derived from adriamycin-resistant breast cancer cells. Biochem. Biophys. Res. Commun..

[153] Wang X., Xu C., Hua Y., Sun L., Cheng K., Jia Z., Han Y., Dong J., Cui Y., Yang Z. (2016). Exosomes play an important role in the process of psoralen reverse multidrug resistance of breast cancer. J. Exp. Clin. Cancer Res..

[154] Pokharel D., Padula M.P., Lu J.F., Jaiswal R., Djordjevic S.P., Bebawy M. (2016). The Role of CD44 and ERM Proteins in Expression and Functionality of P-glycoprotein in Breast Cancer Cells. Molecules.

[155] Torreggiani E., Roncuzzi L., Perut F., Zini N., Baldini N. (2016). Multimodal transfer of MDR by exosomes in human osteosarcoma. Int. J. Oncol..

[156] Zhang F.F., Zhu Y.F., Zhao Q.N., Yang D.T., Dong Y.P., Jiang L., Xing W.X., Li X.Y., Xing H., Shi M. (2014). Microvesicles mediate transfer of P-glycoprotein to paclitaxel-sensitive A2780 human ovarian cancer cells, conferring paclitaxel-resistance. Eur. J. Pharmacol..

[157] Bebawy M., Combes V., Lee E., Jaiswal R., Gong J., Bonhoure A., Grau G.E. (2009). Membrane microparticles mediate transfer of P-glycoprotein to drug sensitive cancer cells. Leukemia.

[158] Levchenko A., Mehta B.M., Niu X., Kang G., Villafania L., Way D., Polycarpe D., Sadelain M., Larson S.M. (2005). Intercellular transfer of P-glycoprotein mediates acquired multidrug resistance in tumor cells. Proc. Natl. Acad. Sci. USA.

[159] Wang X., Qiao D., Chen L., Xu M., Chen S., Huang L., Wang F., Chen Z., Cai J., Fu L. (2019). Chemotherapeutic drugs stimulate the release and recycling of extracellular vesicles to assist cancer cells in developing an urgent chemoresistance. Mol. Cancer.

[160] Ifergan I., Scheffer G.L., Assaraf Y.G. (2005). Novel extracellular vesicles mediate an ABCG2-dependent anticancer drug sequestration and resistance. Cancer Res..

[161] Goler-Baron V., Assaraf Y.G. (2011). Structure and function of ABCG2-rich extracellular vesicles mediating multidrug resistance. PLoS ONE.

[162] Kreger B.T., Johansen E.R., Cerione R.A., Antonyak M.A. (2016). The Enrichment of Survivin in Exosomes from Breast Cancer Cells Treated with Paclitaxel Promotes Cell Survival and Chemoresistance. Cancers (Basel).

[163] de Souza P.S., Cruz A.L., Viola J.P., Maia R.C. (2015). Microparticles induce multifactorial resistance through oncogenic pathways independently of cancer cell type. Cancer Sci..

[164] Khoo X.H., Paterson I.C., Goh B.H., Lee W.L. (2019). Cisplatin-Resistance in Oral Squamous Cell Carcinoma: Regulation by Tumor Cell-Derived Extracellular Vesicles. Cancers.

[165] Zhang Q., Liu R.X., Chan K.W., Hu J., Zhang J., Wei L., Tan H., Yang X., Liu H. (2019). Exosomal transfer of p-STAT3 promotes acquired 5-FU resistance in colorectal cancer cells. J. Exp. Clin. Cancer Res..

[166] Zhao K., Wang Z., Li X., Liu J.L., Tian L., Chen J.Q. (2019). Exosome-mediated transfer of CLIC1 contributes to the vincristine-resistance in gastric cancer. Mol. Cell Biochem..

[167] Guerra F., Paiano A., Migoni D., Girolimetti G., Perrone A.M., De Iaco P., Fanizzi F.P., Gasparre G., Bucci C. (2019). Modulation of RAB7A Protein Expression Determines Resistance to Cisplatin through Late Endocytic Pathway Impairment and Extracellular Vesicular Secretion. Cancers (Basel).

[168] Wu H., Zhou J., Mei S., Wu D., Mu Z., Chen B., Xie Y., Ye Y., Liu J. (2017). Circulating exosomal microRNA-96 promotes cell proliferation, migration and drug resistance by targeting LMO7. J. Cell Mol. Med..

[169] Yu D.D., Wu Y., Zhang X.H., Lv M.M., Chen W.X., Chen X., Yang S.J., Shen H., Zhong S.L., Tang J.H. (2016). Exosomes from adriamycin-resistant breast cancer cells transmit drug resistance partly by delivering miR-222. Tumour. Biol..

[170] Ozawa P.M.M., Alkhilaiwi F., Cavalli I.J., Malheiros D., de Souza Fonseca Ribeiro E.M., Cavalli L.R. (2018). Extracellular vesicles from triple-negative breast cancer cells promote proliferation and drug resistance in non-tumorigenic breast cells. Breast Cancer Res. Treat..

[171] Feng Y., Hang W., Sang Z., Li S., Xu W., Miao Y., Xi X., Huang Q. (2019). Identification of exosomal and nonexosomal microRNAs associated with the drug resistance of ovarian cancer. Mol. Med. Rep..

[172] Mikamori M., Yamada D., Eguchi H., Hasegawa S., Kishimoto T., Tomimaru Y., Asaoka T., Noda T., Wada H., Kawamoto K. (2017). MicroRNA-155 Controls Exosome Synthesis and Promotes Gemcitabine Resistance in Pancreatic Ductal Adenocarcinoma. Sci. Rep..

[173] Patel G.K., Khan M.A., Bhardwaj A., Srivastava S.K., Zubair H., Patton M.C., Singh S., Khushman M., Singh A.P. (2017). Exosomes confer chemoresistance to pancreatic cancer cells by promoting ROS detoxification and miR-155-mediated suppression of key gemcitabine-metabolising enzyme, DCK. Br. J. Cancer.

[174] Wei Y., Lai X., Yu S., Chen S., Ma Y., Zhang Y., Li H., Zhu X., Yao L., Zhang J. (2014). Exosomal miR-221/222 enhances tamoxifen resistance in recipient ER-positive breast cancer cells. Breast Cancer Res. Treat..

[175] Gu Y.Y., Yu J., Zhang J.F., Wang C. (2019). Suppressing the secretion of exosomal miR-19b by gw4869 could regulate oxaliplatin sensitivity in colorectal cancer. Neoplasma.

[176] Akao Y., Khoo F., Kumazaki M., Shinohara H., Miki K., Yamada N. (2014). Extracellular disposal of tumor-suppressor miRs-145 and -34a via microvesicles and 5-FU resistance of human colon cancer cells. Int. J. Mol. Sci..

[177] He J., He J., Min L., He Y., Guan H., Wang J., Peng X. (2020). Extracellular vesicles transmitted miR-31-5p promotes sorafenib resistance by targeting MLH1 in renal cell carcinoma. Int. J. Cancer.

[178] Shiozawa K., Shuting J., Yoshioka Y., Ochiya T., Kondo T. (2018). Extracellular vesicle-encapsulated microRNA-761 enhances pazopanib resistance in synovial sarcoma. Biochem. Biophys. Res. Commun..

[179] Yin J., Zeng A., Zhang Z., Shi Z., Yan W., You Y. (2019). Exosomal transfer of miR-1238 contributes to temozolomide-resistance in glioblastoma. EBioMedicine.

[180] Yuwen D., Ma Y., Wang D., Gao J., Li X., Xue W., Fan M., Xu Q., Shen Y., Shu Y. (2019). Prognostic Role of Circulating Exosomal miR-425-3p for the Response of NSCLC to Platinum-Based Chemotherapy. Cancer Epidemiol. Biomark. Prev..

[181] Ma Y., Yuwen D., Chen J., Zheng B., Gao J., Fan M., Xue W., Wang Y., Li W., Shu Y. (2019). Exosomal Transfer Of Cisplatin-Induced miR-425-3p Confers Cisplatin Resistance In NSCLC Through Activating Autophagy. Int. J. Nanomed..

[182] Wang G., Zhao W., Wang H., Qiu G., Jiang Z., Wei G., Li X. (2019). Exosomal MiR-744 Inhibits Proliferation and Sorafenib Chemoresistance in Hepatocellular Carcinoma by Targeting PAX2. Med. Sci. Monit..

[183] Qin X., Yu S., Zhou L., Shi M., Hu Y., Xu X., Shen B., Liu S., Yan D., Feng J. (2017). Cisplatin-resistant lung cancer cell-derived exosomes increase cisplatin resistance of recipient cells in exosomal miR-100-5p-dependent manner. Int. J. Nanomed..

[184] Cao Y.L., Zhuang T., Xing B.H., Li N., Li Q. (2017). Exosomal DNMT1 mediates cisplatin resistance in ovarian cancer. Cell Biochem. Funct..

[185] Dong H., Wang W., Chen R., Zhang Y., Zou K., Ye M., He X., Zhang F., Han J. (2018). Exosome-mediated transfer of lncRNASNHG14 promotes trastuzumab chemoresistance in breast cancer. Int. J. Oncol..

[186] Takahashi K., Yan I.K., Kogure T., Haga H., Patel T. (2014). Extracellular vesicle-mediated transfer of long non-coding RNA ROR modulates chemosensitivity in human hepatocellular cancer. FEBS Open Biol..

[187] Takahashi K., Yan I.K., Wood J., Haga H., Patel T. (2014). Involvement of extracellular vesicle long noncoding RNA (linc-VLDLR) in tumor cell responses to chemotherapy. Mol. Cancer Res..

[188] Chen Y., Liu L., Li J., Du Y., Wang J., Liu J. (2019). Effects of long noncoding RNA (linc-VLDLR) existing in extracellular vesicles on the occurrence and multidrug resistance of esophageal cancer cells. Pathol. Res. Pract..

[189] Qu L., Ding J., Chen C., Wu Z.J., Liu B., Gao Y., Chen W., Liu F., Sun W., Li X.F. (2016). Exosome-Transmitted lncARSR Promotes Sunitinib Resistance in Renal Cancer by Acting as a Competing Endogenous RNA. Cancer Cell.

[190] Wang J., Lv B., Su Y., Wang X., Bu J., Yao L. (2019). Exosome-Mediated Transfer of lncRNA HOTTIP Promotes Cisplatin Resistance in Gastric Cancer Cells by Regulating HMGA1/miR-218 Axis. Oncol. Targets Ther..

[191] Luo X., Wei J., Yang F.L., Pang X.X., Shi F., Wei Y.X., Liao B.Y., Wang J.L. (2019). Exosomal lncRNA HNF1A-AS1 affects cisplatin resistance in cervical cancer cells through regulating microRNA-34b/TUFT1 axis. Cancer Cell Int..

[192] Zhang Z., Yin J., Lu C., Wei Y., Zeng A., You Y. (2019). Exosomal transfer of long non-coding RNA SBF2-AS1 enhances chemoresistance to temozolomide in glioblastoma. J. Exp. Clin. Cancer Res..

[193] Zheng Z., Chen M., Xing P., Yan X., Xie B. (2019). Increased Expression of Exosomal AGAP2-AS1 (AGAP2 Antisense RNA 1) In Breast Cancer Cells Inhibits Trastuzumab-Induced Cell Cytotoxicity. Med. Sci. Monit..

[194] Jung J.H., Lee M.Y., Choi D.Y., Lee J.W., You S., Lee K.Y., Kim J., Kim K.P. (2015). Phospholipids of tumor extracellular vesicles stratify gefitinib-resistant nonsmall cell lung cancer cells from gefitinib-sensitive cells. Proteomics.

[195] Faict S., Oudaert I., D’Auria L., Dehairs J., Maes K., Vlummens P., de Veirman K., de Bruyne E., Fostier K., Vande Broek I. (2019). The Transfer of Sphingomyelinase Contributes to Drug Resistance in Multiple Myeloma. Cancers (Basel).

[196] Fletcher J.I., Williams R.T., Henderson M.J., Norris M.D., Haber M. (2016). ABC transporters as mediators of drug resistance and contributors to cancer cell biology. Drug Resist. Update.

[197] Gong J., Luk F., Jaiswal R., George A.M., Grau G.E., Bebawy M. (2013). Microparticle drug sequestration provides a parallel pathway in the acquisition of cancer drug resistance. Eur. J. Pharmacol..

[198] Lu J.F., Luk F., Gong J., Jaiswal R., Grau G.E., Bebawy M. (2013). Microparticles mediate MRP1 intercellular transfer and the re-templating of intrinsic resistance pathways. Pharmacol. Res..

[199] Lu J.F., Pokharel D., Bebawy M. (2017). A novel mechanism governing the transcriptional regulation of ABC transporters in MDR cancer cells. Drug Deliv. Transl. Res..

[200] Jaiswal R., Luk F., Dalla P.V., Grau G.E., Bebawy M. (2013). Breast cancer-derived microparticles display tissue selectivity in the transfer of resistance proteins to cells. PLoS ONE.

[201] Ji R., Zhang B., Zhang X., Xue J., Yuan X., Yan Y., Wang M., Zhu W., Qian H., Xu W. (2015). Exosomes derived from human mesenchymal stem cells confer drug resistance in gastric cancer. Cell Cycle.

[202] Mansoori B., Sandoghchian Shotorbani S., Baradaran B. (2014). RNA interference and its role in cancer therapy. Adv. Pharm. Bull..

[203] Zhang H., McCarty N. (2017). Tampering with cancer chemoresistance by targeting the TGM2-IL6-autophagy regulatory network. Autophagy.

[204] Diaz-Hidalgo L., Altuntas S., Rossin F., D’Eletto M., Marsella C., Farrace M.G., Falasca L., Antonioli M., Fimia G.M., Piacentini M. (2016). Transglutaminase type 2-dependent selective recruitment of proteins into exosomes under stressful cellular conditions. Biochim. Biophys. Acta.

[205] Skog J., Wurdinger T., van Rijn S., Meijer D.H., Gainche L., Sena-Esteves M., Curry W.T., Carter B.S., Krichevsky A.M., Breakefield X.O. (2008). Glioblastoma microvesicles transport RNA and proteins that promote tumour growth and provide diagnostic biomarkers. Nat. Cell Biol..

[206] Xiao X., Yu S., Li S., Wu J., Ma R., Cao H., Zhu Y., Feng J. (2014). Exosomes: Decreased sensitivity of lung cancer A549 cells to cisplatin. PLoS ONE.

[207] Sousa D., Matthiesen R., Lima R.T., Vasconcelos M.H. (2020). Deep Sequencing Analysis Reveals Distinctive Non-Coding RNAs When Comparing Tumor Multidrug-Resistant Cells and Extracellular Vesicles with Drug-Sensitive Counterparts. Cancers (Basel).

[208] Janas T., Janas M.M., Sapon K., Janas T. (2015). Mechanisms of RNA loading into exosomes. FEBS Lett..

[209] Kong J.N., He Q., Wang G., Dasgupta S., Dinkins M.B., Zhu G., Kim A., Spassieva S., Bieberich E. (2015). Guggulsterone and bexarotene induce secretion of exosome-associated breast cancer resistance protein and reduce doxorubicin resistance in MDA-MB-231 cells. Int. J. Cancer.

[210] Soekmadji C., Nelson C.C. (2015). The Emerging Role of Extracellular Vesicle-Mediated Drug Resistance in Cancers: Implications in Advanced Prostate Cancer. Biomed. Res. Int..

[211] Hu Y., Yan C., Mu L., Huang K., Li X., Tao D., Wu Y., Qin J. (2015). Fibroblast-Derived Exosomes Contribute to Chemoresistance through Priming Cancer Stem Cells in Colorectal Cancer. PLoS ONE.

[212] Ren J., Ding L., Zhang D., Shi G., Xu Q., Shen S., Wang Y., Wang T., Hou Y. (2018). Carcinoma-associated fibroblasts promote the stemness and chemoresistance of colorectal cancer by transferring exosomal lncRNA H19. Ther. Anostics.

[213] Richards K.E., Zeleniak A.E., Fishel M.L., Wu J., Littlepage L.E., Hill R. (2017). Cancer-associated fibroblast exosomes regulate survival and proliferation of pancreatic cancer cells. Oncogene.

[214] Deng X., Ruan H., Zhang X., Xu X., Zhu Y., Peng H., Zhang X., Kong F., Guan M. (2020). Long noncoding RNA CCAL transferred from fibroblasts by exosomes promotes chemoresistance of colorectal cancer cells. Int. J. Cancer.

[215] Qin X., Guo H., Wang X., Zhu X., Yan M., Wang X., Xu Q., Shi J., Lu E., Chen W. (2019). Exosomal miR-196a derived from cancer-associated fibroblasts confers cisplatin resistance in head and neck cancer through targeting CDKN1B and ING5. Genome Biol..

[216] Au Yeung C.L., Co N.N., Tsuruga T., Yeung T.L., Kwan S.Y., Leung C.S., Li Y., Lu E.S., Kwan K., Wong K.K. (2016). Exosomal transfer of stroma-derived miR21 confers paclitaxel resistance in ovarian cancer cells through targeting APAF1. Nat. Commun..

[217] Fei F., Joo E.J., Tarighat S.S., Schiffer I., Paz H., Fabbri M., Abdel-Azim H., Groffen J., Heisterkamp N. (2015). B-cell precursor acute lymphoblastic leukemia and stromal cells communicate through Galectin-3. Oncotarget.

[218] Wang J., Hendrix A., Hernot S., Lemaire M., de Bruyne E., van Valckenborgh E., Lahoutte T., de Wever O., Vanderkerken K., Menu E. (2014). Bone marrow stromal cell-derived exosomes as communicators in drug resistance in multiple myeloma cells. Blood.

[219] Boelens M.C., Wu T.J., Nabet B.Y., Xu B., Qiu Y., Yoon T., Azzam D.J., Twyman-Saint Victor C., Wiemann B.Z., Ishwaran H. (2014). Exosome transfer from stromal to breast cancer cells regulates therapy resistance pathways. Cell.

[220] Bliss S.A., Sinha G., Sandiford O.A., Williams L.M., Engelberth D.J., Guiro K., Isenalumhe L.L., Greco S.J., Ayer S., Bryan M. (2016). Mesenchymal Stem Cell-Derived Exosomes Stimulate Cycling Quiescence and Early Breast Cancer Dormancy in Bone Marrow. Cancer Res..

[221] Lobb R.J., van Amerongen R., Wiegmans A., Ham S., Larsen J.E., Moller A. (2017). Exosomes derived from mesenchymal non-small cell lung cancer cells promote chemoresistance. Int. J. Cancer.

[222] Challagundla K.B., Wise P.M., Neviani P., Chava H., Murtadha M., Xu T., Kennedy R., Ivan C., Zhang X., Vannini I. (2015). Exosome-mediated transfer of microRNAs within the tumor microenvironment and neuroblastoma resistance to chemotherapy. J. Natl. Cancer Inst..

[223] Muralidharan-Chari V., Kohan H.G., Asimakopoulos A.G., Sudha T., Sell S., Kannan K., Boroujerdi M., Davis P.J., Mousa S.A. (2016). Microvesicle removal of anticancer drugs contributes to drug resistance in human pancreatic cancer cells. Oncotarget.

[224] Dorayappan K.D.P., Wanner R., Wallbillich J.J., Saini U., Zingarelli R., Suarez A.A., Cohn D.E., Selvendiran K. (2018). Hypoxia-induced exosomes contribute to a more aggressive and chemoresistant ovarian cancer phenotype: A novel mechanism linking STAT3/Rab proteins. Oncogene.

[225] Safaei R., Larson B.J., Cheng T.C., Gibson M.A., Otani S., Naerdemann W., Howell S.B. (2005). Abnormal lysosomal trafficking and enhanced exosomal export of cisplatin in drug-resistant human ovarian carcinoma cells. Mol. Cancer Ther..

[226] Shedden K., Xie X.T., Chandaroy P., Chang Y.T., Rosania G.R. (2003). Expulsion of small molecules in vesicles shed by cancer cells: Association with gene expression and chemosensitivity profiles. Cancer Res..

[227] Aubertin K., Silva A.K., Luciani N., Espinosa A., Djemat A., Charue D., Gallet F., Blanc-Brude O., Wilhelm C. (2016). Massive release of extracellular vesicles from cancer cells after photodynamic treatment or chemotherapy. Sci. Rep..

[228] Lopes-Rodrigues V., Di Luca A., Sousa D., Seca H., Meleady P., Henry M., Lima R.T., O’Connor R., Vasconcelos M.H. (2016). Multidrug resistant tumour cells shed more microvesicle-like EVs and less exosomes than their drug-sensitive counterpart cells. Biochim. Biophys. Acta.

[229] Goler-Baron V., Sladkevich I., Assaraf Y.G. (2012). Inhibition of the PI3K-Akt signaling pathway disrupts ABCG2-rich extracellular vesicles and overcomes multidrug resistance in breast cancer cells. Biochem. Pharmacol..

[230] Yin J., Yan X., Yao X., Zhang Y., Shan Y., Mao N., Yang Y., Pan L. (2012). Secretion of annexin A3 from ovarian cancer cells and its association with platinum resistance in ovarian cancer patients. J. Cell Mol. Med..

[231] Yan X., Yin J., Yao H., Mao N., Yang Y., Pan L. (2010). Increased expression of annexin A3 is a mechanism of platinum resistance in ovarian cancer. Cancer Res..

[232] Ciravolo V., Huber V., Ghedini G.C., Venturelli E., Bianchi F., Campiglio M., Morelli D., Villa A., Della Mina P., Menard S. (2012). Potential role of HER2-overexpressing exosomes in countering trastuzumab-based therapy. J. Cell Physiol..

[233] Guerra F., Bucci C. (2019). Role of the RAB7 Protein in Tumor Progression and Cisplatin Chemoresistance. Cancers (Basel).

